# Continuous versus Standard Palbociclib Treatment and Molecular Profiling of Solid Tissues and Liquid Biopsies in the CCTG MA.38 Trial in Advanced Breast Cancer

**DOI:** 10.1158/2767-9764.CRC-25-0346

**Published:** 2025-11-13

**Authors:** Anil Abraham Joy, Nicholas Cheng, Karen A. Gelmon, Mihaela Mates, Christine Desbiens, Mark Clemons, Sara Taylor, Julie Lemieux, Angela DeLuca, Louis Gasparini, Ilinca Lungu, David Soave, Alex Fortuna, Trevor Pugh, Shuo Shuo Liu, John M.S. Bartlett, Philip Awadalla, Melanie Spears, Bingshu E. Chen, Jane Bayani, Wendy R. Parulekar

**Affiliations:** 1 Division of Medical Oncology, Department of Oncology, Cross Cancer Institute, University of Alberta, Edmonton, Canada.; 2 Computational Biology, Ontario Institute for Cancer Research, Toronto, Canada.; 3 Department of Molecular Genetics, University of Toronto, Toronto, Canada.; 4 Department of Medical Oncology, Vancouver Centre, BC Cancer, Vancouver, Canada.; 5 Department of Oncology, Queen’s University, Kingston Health Sciences Centre, Kingston, Canada.; 6 Centre hospitalier universitaire de Québec-Université Laval, Québec, Canada.; 7 Division of Medical Oncology, Ottawa Hospital Research Institute, University of Ottawa, Ottawa, Canada.; 8 Medical Oncology, BC Cancer Agency, Kelowna, Canada.; 9 Centre hospitalier universitaire de Québec-Université Laval, Université Laval Cancer Research Center, Québec, Canada.; 10 Diagnostic Development, Ontario Institute for Cancer Research, Toronto, Canada.; 11 Department of Mathematics, Wilfrid Laurier University, Waterloo, Canada.; 12 Genome Sequence Informatics, Ontario Institute for Cancer Research, Toronto, Canada.; 13 Department of Medical Biophysics, University of Toronto, Toronto, Canada.; 14 Princess Margaret Hospital, University Health Network, Toronto, Canada.; 15 Department of Statistics and Actuarial Sciences, University of Waterloo, Waterloo, Canada.; 16 Edinburgh Cancer Research Centre, Western General Hospital, Edinburgh, United Kingdom.; 17 Dalla Lana School of Public Health, University of Toronto, Toronto, Canada.; 18 Nuffield Department of Population Health, Big Data Institute, University of Oxford, Oxford, United Kingdom.; 19 Department of Laboratory Medicine and Pathobiology, University of Toronto, Toronto, Canada.; 20 Canadian Cancer Trials Group, Queen’s University, Kingston, Canada.

## Abstract

The randomized phase II MA.38 trial estimated the relative progression-free survival (PFS) associated with second-line endocrine therapy plus palbociclib administered on a 100 mg continuous daily dosing (CDD) schedule compared with the standard dose schedule (SDS) of 125 mg (days 1–21 of a 28-day cycle). A total of 180 patients were allocated 1:1 to protocol therapy. Molecular profiling was performed on the archival tissue and cell-free DNA (cfDNA) at enrollment, 3 months, and 6 months. The primary analysis for PFS demonstrated a similar outcome for the CDD versus SDS treatment strategy: HR = 0.93 (90% confidence interval, 0.66–1.30). Secondary efficacy measures for CDD versus SDS included the following: overall survival, HR = 1.07 (90% confidence interval, 0.67–1.69); response rate, 16.1% versus 18.0% (*P* = 0.66); median duration of response, 4.2 months (range, 2.8–13.9 months) versus 5.6 months (range, 2.4–13.9 months; *P* = 0.86); and clinical benefit rate, 53.2% versus 57.3% (*P* = 0.89). cfDNA profiling of the baseline enrollment sample prior to palbociclib commencement showed low tumor fraction (HR = 2.28; *P* = 9.9 × 10^−6^); higher short/long fragment length ratios (HR = 1.19; *P* = 0.049) and cfDNA variants in *FGFR4* (HR = 3.65; *P* = 0.012) were prognostic and associated with inferior PFS. Variants in *TP53* (HR = 2.48; *P* = 0.006) and ESR1 (HR = 3.42; *P* = 0.005) detected at 12 weeks on treatment were also associated with poor PFS. CDD palbociclib 100 mg dosing was not associated with improved efficacy compared with the standard intermittent 125 mg dosing schedule. Additionally, we identified prognostic biomarkers in alignment with prior research and demonstrated the value of cfDNA dynamics, including fragment length ratios and tumor fraction as a measure of treatment response.

**Significance:**

A continuous 100 mg dosing schedule of palbociclib was tolerable but not associated with improved efficacy signals versus the standard intermittent 125 mg (days 1–21 of a 28-day cycle) schedule. Mutations detected in liquid biopsies and changes in cfDNA dynamics were linked to poor outcomes and may identify patients with treatment-resistant cancer.

## Introduction

Endocrine therapy (ET) has resulted in major improvements in outcomes for patients with estrogen receptor–positive (ER^+^) breast cancer (1). Nonetheless, many patients experience disease relapse or progression due to therapy resistance (2–4). In palbociclib and other CDK4/6 inhibitor trials, improved patient outcomes were observed (5), resulting in CDK4/6 inhibitors plus ET being the standard of care for advanced hormone receptor–positive (HR+) breast cancer treatment.

Phase I data testing a 21-day of a 28-day palbociclib dosing schedule identified 125 mg as the recommended phase II dose (standard dose schedule, SDS), with uncomplicated grade 3 or 4 neutropenia as the dose-limiting toxicity (6). This dose has been shown to be efficacious and safe in phase II and phase III trials evaluating palbociclib in combination with ET compared with et alone (7–11). Cumulative clinical data suggest that a significant number of patients treated with palbociclib experience dose interruptions, delays, and reductions due to neutropenia without apparent detriment in disease outcomes compared with the SDS (12, 13). Preclinical studies suggested that palbociclib exerts an antiproliferative effect on cancer cells that is reversed upon drug discontinuation, and that continuous dosing could be as efficacious as intermittent dosing. Pharmacodynamic and pharmacokinetic models to predict neutropenia based on the palbociclib dosing schedule indicated that neutropenia is dose-dependent, rapidly reversible, and noncumulative (14). Therefore, we hypothesized that the 100 mg continuous daily dose (CDD) of palbociclib may be more active and associated with fewer instances of grade 3 and 4 neutropenia or febrile neutropenia compared with the SDS.

We now report on the MA.38 trial dosing regimen efficacy, the molecular profiling of banked treatment-naïve tissue retrieved from the initial presentation of breast cancer and of the cell-free DNA (cfDNA) at trial enrollment and at 12 and 24 weeks on study.

## Patients and Methods

### Study design and patients

MA.38 (NCT02630693) was a pan-Canadian, open-label, multicenter, randomized phase II trial led by the Canadian Cancer Trials Group (CCTG) to estimate the efficacy of orally administered 100 mg palbociclib on a CDD schedule compared with 125 mg on an SDS schedule (i.e., 125 mg on days 1–21 of each 28-day cycle) with the physician’s choice of ET. Female patients 18 years of age or older, with advanced or metastatic, histologically confirmed adenocarcinoma of the breast with ER^+^, any progesterone receptor status, and HER2-negative (HER2^−^) status were eligible. Other key eligibility criteria included the discontinuation of ET due to disease progression either during or within 1 month for advanced/metastatic disease or during or within 12 months of completion of adjuvant ET, a maximum of one line of chemotherapy for advanced/metastatic disease, evidence of disease on imaging studies, Eastern Cooperative Oncology Group (ECOG) score of 0 to 2, and adequate bone marrow and organ function. Patients were not eligible if they had advanced, symptomatic, and visceral disease of immediate/life-threatening risk; symptomatic central nervous system, meningeal, or parenchymal brain involvement that was uncontrolled or required steroids; or prior treatment with any CDK4/6 or mTOR inhibitor. All enrolled patients provided signed, informed consent to participate in the trial. Other eligibility criteria are described in Supplementary File S1. The trial was conducted under a Clinical Trial Application with Health Canada, complied with Division 5 of the Canadian Regulations Respecting Food and Drugs, and was performed in accordance with International Conference on Harmonization Good Clinical Practice guidelines. The protocol was approved by the local Research Ethics Boards of each participating center. Written informed consent was obtained from the patients for entry into the trial. Trial conduct and toxicity data were reviewed on a real-time basis by the CCTG Statistical and Operations Centre and on a 6-monthly basis by the independent CCTG Data Safety Monitoring Committee. Translational studies were performed at the Ontario Institute of Cancer Research (OICR) with ethics approval from the University of Toronto Research Ethics Board (protocol 36239) and conducted in accordance with guidelines set by the Declaration of Helsinki.

### Study treatment and assessments

Eligible patients were randomized 1:1 to the orally administered palbociclib CDD arm (100 mg daily for each 28-day cycle) or the SDS arm (125 mg on days 1–21 of each 28-day cycle) in combination with investigator choice of ET. Allocation was assigned using a minimization procedure that balanced allocation between arms for the following factors: clinical center, presence of visceral metastases (yes vs. no), previous duration of the most recent ET prior to randomization (≥6 months vs. <6 months in the advanced/metastatic setting or ≥24 months vs. <24 months in the adjuvant setting), and planned ET using fulvestrant, tamoxifen, or aromatase inhibitors (AI). Palbociclib was taken orally with food and combined with ET consisting of one of the following: fulvestrant [500 mg intramuscular injection on days 1 and 15 of a 28-day cycle (for cycle 1) and then day 1 of subsequent 28-day cycles], tamoxifen (20 mg orally daily for a 28-day cycle), or an AI daily for a 28-day cycle [letrozole (2.5 mg orally), anastrozole (1 mg orally), or exemestane (25 mg orally)]. All premenopausal patients required concurrent ovarian suppression if treated with fulvestrant or an AI, but this was optional for premenopausal patients treated with tamoxifen. Dose modifications for hematologic and nonhematologic adverse events were mandated but differed between arms, including mid-cycle, uncomplicated grade 3 neutropenia or thrombocytopenia. In the CDD arm, mid-cycle grade 3 neutropenia or thrombocytopenia mandated an immediate and permanent dose reduction of one level (i.e., to 75 mg), with further modification to an intermittent SDS of 3 out of 4 weeks’ daily dose if recurrent. In contrast, no dose adjustment was required for mid-cycle uncomplicated grade 3 neutropenia or thrombocytopenia in the intermittent SDS treatment arm (see Supplementary File S1). Investigator assessment of tumor response was performed every 12 weeks until disease progression using the RECIST version 1.1 criteria (15).

Adverse events were assessed after each cycle and reported according to the NCI Common Terminology Criteria for Adverse Events version 4.0. A detailed safety evaluation was performed after the initial 12 patients on both arms were enrolled and followed for 8 weeks to assess safety, tolerability, and compliance with therapy, including dose reductions and dose discontinuations. Quality of life (QOL) was measured with the European Organisation for Research and Treatment of Cancer (EORTC) QLQ-C30 supplemented by a trial-specific checklist addressing key symptoms (mouth sores and neuropathy). Questionnaires were administered at baseline, 4, 8, and 12 weeks and then every 12 weeks until progressive disease. Additional details are described in Supplementary File S1. Solid tissues (treatment-naïve) were collected prior to first-line ET. Liquid biopsies were collected at the time of entry into the clinical trial, serving as a baseline, and then every 12 weeks until disease progression. Treatment continued until disease progression, unacceptable toxicity, intercurrent illness requiring discontinuation of protocol therapy, or withdrawal of patient consent.

### Tissue handling and molecular profiling

Archival formalin-fixed, paraffin-embedded tissues, representing the treatment-naïve diagnostic sample, were obtained from 167 out of 180 (93%) patients. Hematoxylin and eosin–stained slides were reviewed, and unstained sections were macrodissected, and nucleic acids were extracted at the OICR (16). Gene expression profiling was performed using 300 ng of RNA against the Human nCounter Breast Cancer 360 (BC360) panel according to the manufacturer’s instructions (NanoString Technologies Inc.). The 778-gene panel contains several proprietary signatures (Supplementary Table S1), including the genomic risk as calculated by the PAM50 signature (17, 18) and the tumor inflammation signature (TIS; ref. 19). Proprietary signature results were generated by NanoString Technologies, with further analyses performed at the OICR. For mutational profiling of genomic DNA from solid tumor DNA, cases with a minimum of 500 ng of DNA were sent to Memorial Sloan Kettering (MSK) for profiling against the Integrated Mutation Profiling of Actionable Cancer Targets panel (MSK; ref. 20). cfDNA was recovered from plasma samples using the QIAamp Circulating Nucleic Acid Kit (QIAGEN Inc.). Targeted cfDNA mutational profiling was performed on 406 plasma samples across 178 patients at baseline (*n* = 170), week 12 (*n* = 160), and week 24 (*n* = 76). The OICR-MA.38 panel is a laboratory-developed panel for somatic mutations within cfDNA and tumor genomic DNA, comprising 94 genes (Supplementary File S1). Sequencing libraries were prepared using the KAPA HyperPrep Kit (Roche Ltd.). Paired-end sequencing was performed using the NextSeq 550 technology (2 × 75 PE; Illumina Inc.). Read alignments were performed using bwa-mem (0.7.12) against the reference genome hg38/p12. Unique molecule-based error suppression was completed with ConsensusCruncher (https://github.com/pughlab/ConsensusCruncher). Single-nucleotide variants and insertion-deletions were called using Mutect2 (GATK 4.1.1.0), subset to the OICR-MA.38 target region and annotated with Variant Effect Predictor (92.0). To suppress sequencing errors and artifacts encountered when performing deep coverage sequencing (i.e., 5,000–20,000×) for the detection of low-frequency variants, the targeted sequencing libraries integrated unique molecular indexes. From raw uncollapsed reads, duplicate reads were amalgamated into single-strand consensus sequences and combined into duplex consensus sequences. Singletons were corrected and combined with single-strand consensus sequences and collapsed into unique molecules using the ConsensusCruncher algorithm (21).

### Main trial statistical analyses

The primary endpoint of the trial was progression-free survival (PFS). Given the lack of clinical data surrounding the efficacy of the 100 mg CDD to inform a target HR for superiority and the need to limit exposure of the new dose schedule to a large number of patients, we chose a methodology that would allow us to estimate the relative efficacy between CDD and SDS palbociclib, which did not mandate a formal test of superiority or noninferiority between the two arms. Based on the PALOMA-3 (NCT 01942135) interim analysis results (9), we estimated the PFS for the 125 mg SD arm would be 10 months. The sample size was based on estimating the HR of the two arms within a 90% confidence interval (CI). We targeted an observed rate of approximately 58 PFS events in each arm to enable the estimation of the upper bound of the 90% CI, which would be 1.36 times the estimated HR, and the lower bound 0.74 times the estimated HR. Assuming a median PFS of approximately 10 months for both treatment arms, a duration of accrual and follow-up of 1 year, and a dropout rate of 10%, 90 subjects in each arm were required. Secondary objectives included the evaluation of safety and tolerability and other measures of efficacy, including overall survival (OS), response rate, duration of response (DOR), clinical benefit rate, and QOL.

Analysis of pretreatment characteristics and all efficacy outcomes is based on the intent-to-treat population. The primary endpoint of PFS was defined as the time from randomization to progression or death from any cause. Patients without a PFS event at the final analysis were censored on the date of the last disease assessment. OS was calculated from the date of randomization to the date of death or was censored at the last date the patient was known to be alive. The PFS and OS differences between the arms were estimated using an HR stratified by the stratification factors at randomization (excluding center). An unstratified multivariable Cox regression model with age, ECOG PS, histology, and grade as covariates was fitted for both analyses. For patients with RECIST version 1.1 criteria, measurable disease, complete response (CR) or partial response (PR), and duration of response were calculated from the time of CR or PR (whichever was first recorded) until the first date of recurrent or progressive disease documentation or death. Duration of response in patients without documentation of progression or death at the time of the clinical cutoff date was censored on the date of the last disease assessment. The difference in duration of response between the two treatment arms was tested using the log-rank test, adjusting for stratification factors at randomization. Safety analyses included data from all patients who received at least one dose of protocol therapy. A Fisher exact test was used to compare the toxicity rates between the treatment arms based on the safety population.

The EORTC QLQ-C30 questionnaire was scored using standard methodology, and a trial-specific checklist was also administered to patients relating to symptoms of sore mouth and neuropathy. QOL analyses were based on the intent-to-treat population; a change score of 10 points from baseline was defined as clinically relevant (22). Distributions of three categories (improved, stable, or worse) between the two arms were compared using a *χ*^2^ test. Statistical software used for all analyses was SAS version 9.2 (SAS Institute Inc.).

### Molecular and informatic analyses

#### Gene expression and PFS

For the association between gene expression levels in the treatment-naïve solid tumor samples and PFS, samples were stratified according to expression levels above and below the cohort median per gene. A univariate Cox proportional hazards (PH) model was fit using high or low expression of individual genes as the predictor variable to calculate HR, 95% CI, and log-rank test *P* values. Statistical significance was corrected for multiple testing by calculating the FDR.

#### Association between gene expression signatures and PFS

Samples were stratified according to whether their gene expression signature score was above or below the cohort median. The association between the overall PFS and individual gene expression signatures was calculated by fitting a univariate Cox PH model to calculate HR, 95% CI, and log-rank test *P* values using a signature score above and below the cohort median as the predictor variable. *P* values were adjusted for multiple testing by calculating the FDR.

#### Association between the presence of somatic variants at baseline and week 12 cfDNA samples and PFS

As paired peripheral blood leukocytes were not profiled, potential germline variants were removed by selecting variants with a maximum variant allele frequency (VAF) of less than 40% and variants with a population allelic frequency in the Genome Aggregation Database and Exome Aggregation Consortium of less than 1%. All silent or synonymous and untranslated region mutations were removed to exclude passenger mutations, only retaining splice site, missense, nonsense, frameshift, and in-frame insertions and deletions. Single-nucleotide variants were only considered if located in exons or splice site regions with a minimum alternative allele depth of 5 and an overall depth of 100 at the same position. All downstream analyses were performed using the remaining protein-coding and splice site variants. The association between overall PFS and the presence of genomic aberrations in blood samples collected at baseline and at week 12 across individual driver genes was assessed by fitting univariate Cox PH models, with the presence or absence of genomic aberrations in the tested gene as the primary predictor to calculate HR, 95% CIs, and log-rank *P* values. *P* values were considered significant for values less than 0.05.

#### Association between tumor fraction and PFS

Tumor fraction was estimated by computing the maximum VAF across all genomic aberrations located at exons and splice site regions detected within each sample at baseline and at week 12. The change in tumor fraction was calculated by subtracting the maximum baseline VAF from the maximum week 12 VAF. The association between baseline or week 12 tumor fraction and PFS was investigated by stratifying samples above and below varying cutoffs between 1% and 20%. Likewise, the association between PFS and change in tumor fraction was also assessed at varying cutoffs from 28% to 30%. The HR, 95% CI, and log-rank *P* values at each tumor fraction cutoff and tumor fraction change cutoff were calculated by fitting a univariate Cox PH model as described above, using a VAF above or below the cutoff as the predictor variable.

#### Association between fragment size ratios and PFS

cfDNA fragment sizes were calculated from estimated insert sizes across paired-end reads in each sample from BAM files. Varying fragment size cutoffs between 135 and 180 base pairs were considered when stratifying fragments into long (above cutoff) or short (below cutoff) fragments (23, 24). The short/long fragment ratio was calculated by dividing the total number of short fragments by the number of long fragments per participant. The association between the short/long fragment ratio and overall PFS was assessed by fitting a univariate Cox PH model using the fragment ratio above and below the cohort median as the predictor variable to calculate HR, 95% CI, and log-rank *P* values. We additionally calculated the correlation between short/long fragment ratios and overall PFS across samples with an event by performing a Pearson’s correlation.

### Gene set enrichment analysis

Gene set enrichment analysis to identify significantly enriched biological processes, molecular functions, and Reactome pathways was performed using gene ontology enrichment analysis (https://geneontology.org/).

### Visualization tools

All heat maps and statistical analyses were performed using R (version 4.0.3). Cox PH models were conducted using the *survival* (R package version 3.5-3). Fisher’s exact tests were performed using *stats* (R package version 3.6.2). Kaplan–Meier plots were generated using *survminer* (R package version 0.4.9). Remaining plots, including heatmaps, bar plots, and scatter plots, were generated using *ggplot2* (R package version 3.4.4).

## Results

### Patient accrual and treatment

**Figure 1.**
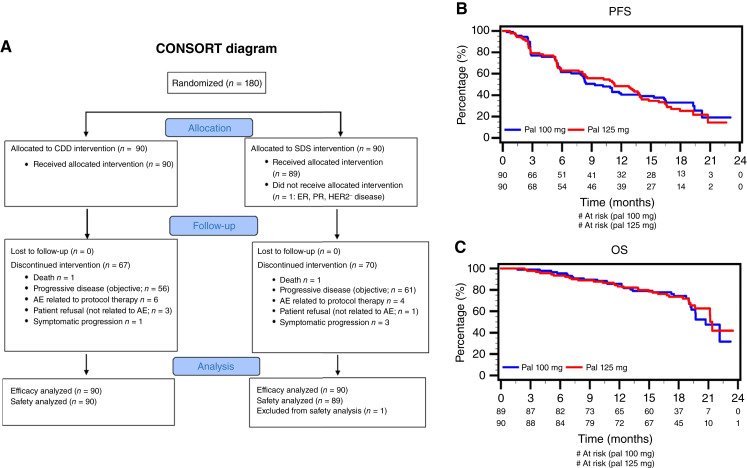
MA.38 main trial results. **A,** CONSORT diagram. **B** and **C,** Results from the MA.38 trial for (**B**) PFS and (**C**) OS among patients in the CDS (blue) and SDS (red). AE, adverse event.

Between December 2015 and February 2017, 180 patients were enrolled across 20 centers in Canada (25) and received ET plus palbociclib, either as 100 mg CDD (*n* = 90) or 125 mg SDS (*n* = 89; Fig. 1A). After a median follow-up of 19.0 months (range, 1.7–26.7 months), 121 PFS events were documented, thus meeting the protocol requirement of a minimum of 116 events to trigger the final analysis. The clinical cutoff date for the final analysis was April 16, 2018, with a database lock on August 8, 2018, for the final analysis.

Baseline characteristics for the whole population included the following: median age 60.5 years (21% > 70 years), 95% of patients with ECOG PS 0/1, 91% with postmenopausal status, 67% with visceral metastases, and 31% with grade 3 tumors. The planned ET was AIs in 33%, fulvestrant in 57%, and tamoxifen in 10% of patients (Table 1). Patients in the CDD group received a median daily dose of 87 mg compared with 125 mg in the SDS group. Similarly, the cumulative dose of palbociclib was lower in the CDD versus the SDS arm: 23,629 mg (range, 500–66,600 mg) versus 26,430 mg (range, 2,250–60,250 mg; Supplementary Table S2). The dose intensity for the CDD group was 555.7 mg/week, and for the SDS group, it was 580.8 mg/week. Higher rates of dose reductions in the CDD schedule were observed (68.9% vs. 28.1%) due to neutropenia. Dose discontinuation related to adverse events associated with the therapy was 6.7% versus 4.5% in the CDD versus the SDS arm, respectively.

**Table 1.** Baseline characteristics.

Patient/disease characteristic	Number of patients (%)					
	Palbociclib 100 mg (*N* = 90)		Palbociclib 125 mg (*N* = 90)		Total (*N* = 180)	
Age	​	​	​	​	​	​
Median (IQR)	61.5	(53.0–69.0)	59.5	(53.0–69.0)	60.5	(53.0–69.0)
≤39	2	(2.2)	3	(3.3)	5	(2.8)
40–49	11	(12.2)	9	(10.0)	20	(11.1)
50–59	26	(28.9)	33	(36.7)	59	(32.8)
60–69	35	(38.9)	24	(26.7)	59	(32.8)
≥70	16	(17.8)	21	(23.3)	37	(20.6)
Race	​	​	​	​	​	​
Asian	10	(11.1)	6	(6.7)	16	(8.9)
Black or African American	1	(1.1)	1	(1.1)	2	(1.1)
American Indian or Alaska Native	0	(0.0)	2	(2.2)	2	(1.1)
Native Hawaiian or Pacific Islander	1	(1.1)	0	(0.0)	1	(0.6)
Not reported, refused, or unknown	2	(2.2)	6	(6.7)	8	(4.4)
White	76	(84.4)	75	(83.3)	151	(83.9)
ECOG PS	​	​	​	​	​	​
0	48	(53.3)	32	(35.6)	80	(44.4)
1	37	(41.1)	54	(60.0)	91	(50.6)
2	5	(5.6)	4	(4.4)	9	(5.0)
Menopausal status	​	​	​	​	​	​
Postmenopausal	83	(92.2)	80	(88.9)	163	(90.6)
Pre- or perimenopausal	7	(7.8)	10	(11.1)	17	(9.4)
Visceral metastases	​	​	​	​	​	​
Yes	60	(66.7)	60	(66.7)	120	(66.7)
No	30	(33.3)	30	(33.3)	60	(33.3)
Duration of exposure to prior ET	​	​	​	​	​	​
≥6 Months (advanced)	51	(56.7)	47	(52.2)	98	(54.4)
<6 Months (advanced)	16	(17.8)	14	(15.6)	30	(16.7)
≥24 Months (adjuvant)	14	(15.6)	18	(20.0)	32	(17.8)
<24 Months (adjuvant)	9	(10.0)	11	(12.2)	20	(11.1)
Planned ET	​	​	​	​	​	​
AI	30	(33.3)	30	(33.3)	60	(33.3)
Fulvestrant	51	(56.7)	51	(56.7)	102	(56.7)
Tamoxifen	9	(10.0)	9	(10.0)	18	(10.0)
Histology	​	​	​	​	​	​
Ductal	78	(86.7)	77	(85.6)	155	(86.1)
Lobular	12	(13.3)	12	(13.3)	24	(13.3)
Missing	0	(0.0)	1	(1.1)	1	(0.6)
PR status	​	​	​	​	​	​
Positive	79	(87.8)	72	(80.0)	151	(83.9)
Negative	11	(12.2)	18	(20.0)	29	(16.1)
Grade	​	​	​	​	​	​
1	13	(14.4)	16	(17.8)	29	(16.1)
2	44	(48.9)	43	(47.8)	87	(48.3)
3	28	(31.1)	28	(31.1)	56	(31.1)
Unknown	5	(5.6)	3	(3.3)	8	(4.4)
Sex	​	​	​	​	​	​
Female	90	(100)	90	(100)	180	(100)
Male	0	(0)	0	(0)	0	(0)

### Efficacy

The HR for PFS in the CDD arm relative to SDS was 0.93 (90% CI, 0.66–1.30; Fig. 1B). The median PFS was 9.3 months in the CDD group (90% CI, 6.9–13.9 months) versus 11.3 months in the SDS group (90% CI, 8.1–13.8 months). Multivariable efficacy results adjusted for predefined clinical factors (age, ECOG, histology, and tumor grade) were consistent with the overall PFS results (HR = 0.92; 90% CI, 0.67–1.25). Grade 2 or 3 tumors were significantly associated with worse PFS compared with grade 1 (HR = 1.95; 90% CI, 1.20–3.19; Table 2).

**Table 2.** PFS and OS.

​	PFS		OS	
	Univariate HR (90% CI)	Multivariable HR (90% CI)^a^	Univariate HR (90% CI)	Multivariable HR (90% CI)^a^
Treatment (100 vs. 125 mg; stratified)	0.93 (0.66, 1.30)	—	1.07 (0.67, 1.69)	—
Treatment (100 vs. 125 mg; unstratified)	0.95 (0.70, 1.28)	0.92 (0.67, 1.25)	1.10 (0.72, 1.68)	1.14 (0.75, 1.75)
ECOG PS (0 vs. 1 or 2)	​	0.91 (0.66, 1.24)	​	1.47 (0.95, 2.30)
Age (<60 vs. ≥60)	​	0.73 (0.48, 1.13)	​	0.94 (0.51, 1.73)
Histology (lobular vs. ductal)	​	1.05 (0.67, 1.64)	​	1.07 (0.58, 1.98)
Tumor grade (2 or 3 vs. 1)	​	1.95 (1.20, 3.19)	​	1.32 (0.73, 2.41)

a Based on Cox model with all factors included.

The HR for OS in the CDD arm relative to SDS was 1.07 (90% CI, 0.67–1.69; Fig. 1C). The median OS was 20.7 months in the CDD group (90% CI, 19.3–23.3 months) versus 21.4 months in the SDS group (90% CI, 19.7–26.7 months). Similar trends were seen in patients with measurable disease (CDD vs. SDS): response rate, 16.1% (*n* = 62) versus 18.0% (*n* = 61; *P* = 0.66); median duration of response (CR or PR), 4.2 months (range, 2.8–13.9 months) versus 5.6 months (range, 2.4–13.9 months; *P* = 0.86); and clinical benefit rate, 38.9% versus 41.6% (*P* = 0.79).

### Toxicity and QOL

The adverse event profiles, regardless of causality, were similar between the two treatment schedules. Adverse events with a frequency of 10% or greater are listed in Supplementary Table S3. Neutropenia was observed more frequently in the CDD schedule (70%) relative to the SDS schedule (40%), with three patients in each arm experiencing febrile neutropenia (Table 2). One patient in each arm died while on protocol therapy due to sepsis following treatment-related febrile neutropenia on the CDD arm and progressive disease on the SDS arm. QOL, as determined by EORTC QLQ-C30 scores and trial specific questions, was not clinically or significantly different between the two treatment schedules.

### Targeted genomic and transcriptomic profiling identifies molecular markers of palbociclib resistance in treatment-naïve solid tumors

**Figure 2.**
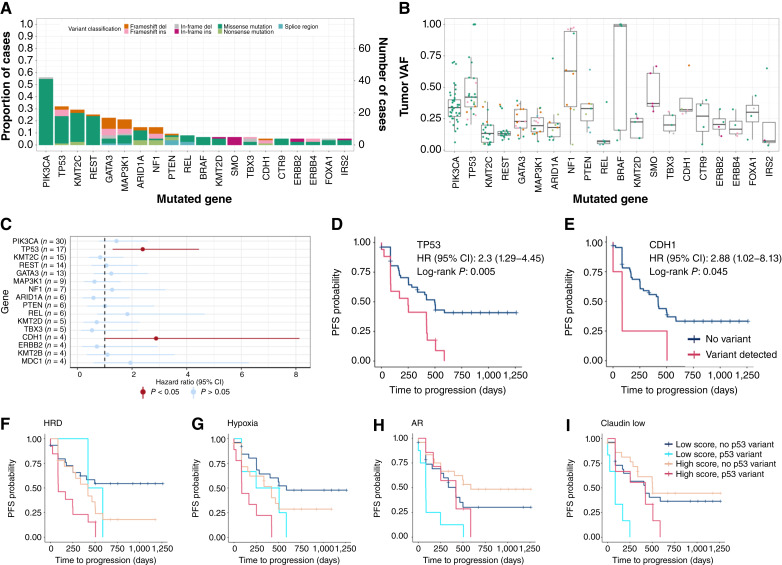
Pretreatment diagnostic tumor somatic variants and transcriptional signatures associated with PFS. **A,** Frequency of variants across breast cancer driver genes in pretreatment tumor biopsies. Colors indicate variant classification (**B**) VAF of detected single-nucleotide variants across samples. **C,** HRs of PFS associated with the presence of somatic variants in driver genes. **D–F,** Kaplan–Meier curves of genetic variants associated with shorter PFS time in (**D**) *TP53* and (**E**) *CDH1*. **F–I,** Kaplan–Meier curves of transcriptional signatures stratified by *TP53* variant status associated with shorter PFS time for (**F**) HRD, (**G**) hypoxia, (**H**) androgen receptor, and (**I**) claudin low.

Treatment-naïve solid tissues/biopsies were available for 167 out of 180 (93%) patients. Ninety-four patient specimens (94/167, 56%) yielded sufficient RNA for gene expression profiling, whereas 76 (76/167, 45%) provided sufficient gDNA for targeted sequencing for the 505 genes in the MSK Integrated Mutation Profiling of Actionable Cancer Targets panel (Supplementary Fig. S1). Nonsilent variants were detected in 75 out of 76 tumors across 195 genes. Mutations in the *PIK3CA* gene were most frequent at 55.3%, followed by *TP53* (31.6%), *KMT2C* (28.9%), *REST* (25.0%), *GATA3* (22.4%), and *MAP3K1* (21.1%; Fig. 2A and B; Supplementary Fig. S2). PAM50 molecular classification of pretreatment tumors revealed that 54 (57%) were luminal B, 36 (38.7%) were luminal A, 2 (2.15%) were basal-like, and 2 (2.15%) were HER2-enriched (HER2-E; Supplementary Fig. S3).

To identify molecular markers of prognostic importance in pretreatment tumors, univariate Cox regression analysis was performed among genes with at least 5% of cases harboring a variant. Only inferred deleterious variants detected in *TP53* (HR = 2.3; log-rank *P* = 0.005) and *CDH1* (HR = 2.88; log-rank *P* = 0.045) were significantly associated with shorter PFS (Fig. 2C–E). Among the 24 samples with a mutation in *TP53* or CDH1, only one case harbored a mutation in more than one of these genes (Supplementary Fig. S2). Most tumors in this study harboring *TP53* mutations were of the luminal B subtype (Supplementary Fig. S2). As in recent reports (26), we found that TP53 mutations were associated with poor PFS across both treatment arms (Fig. 2D). Although *CDH1* mutations were observed at a relatively low frequency in the cohort, we found PFS to be less than 16.42 months among these patients (Fig. 2E), suggesting that mutations in these genes may be prognostic in the study population.

Using the gene signatures and individual gene expression levels, we identified transcriptomic profiles in pretreatment tumors associated with poor PFS when treated with combination palbociclib and ET (Supplementary Figs. S3–S5). Of the 758 genes profiled, 40 genes with high expression and 23 genes with low expression were associated with poor PFS (Supplementary Fig. S4A–S4D). Gene set enrichment analysis indicated that these genes were associated with the cell cycle and cell division, including G_2_–M transition, mitotic spindle assembly, and chromatid segregation (Supplementary Fig. S4E); however, after multiple testing and FDR correction, these genes were not significant. Using the BC360 customized gene expression signatures, we identified seven signatures putatively prognostic. Specifically, androgen receptor (HR = 0.55; *P* = 0.02), progesterone receptor (HR = 0.56; *P* = 0.027), CDK4 (HR = 1.68; *P* = 0.045), breast cancer proliferation (HR = 1.74; *P* = 0.034), breast cancer p53 (HR = 1.77; *P* = 0.029), genomic risk (HR = 1.85; *P* = 0.019), and homologous repair deficiency (HRD; HR = 1.9; *P* = 0.015) expression signatures were significantly associated with PFS (Supplementary Fig. S5).

### Stratification by TP53 mutational status and transcriptional signatures in treatment-naïve diagnostic tumor tissues identifies pathways and features associated with shorter PFS

As *TP53* mutations and a transcriptional HRD signature were found to be independently associated with poor prognosis, we investigated whether transcriptomic signatures stratified by *TP53* status could be more prognostic of PFS following palbociclib treatment (Fig. 2F–I; Supplementary Fig. S6). Cases with low HRD signature scores (below the cohort median) and no TP53 variants experienced overall improved PFS compared with those with high HRD signature scores and *TP53* mutations (Fig. 2G; Supplementary Fig. S6). This was recapitulated with the hypoxia and IDO1 gene expression signature, and in these cases, the presence of a high signature score (above the cohort median) with no *TP53* variant demonstrated intermediate PFS (Fig. 2G; Supplementary Fig. S6). In contrast, patients with high androgen receptor and claudin-low subtype signatures with no *TP53* mutations experienced better PFS compared with those with low signature scores and positive *TP53* mutations (Fig. 2H and I; Supplementary Fig. S6). Individuals harboring *TP53* mutations and a high macrophage, mammary stemness, or stroma gene expression signature also exhibited PFS of less than 8.5 months following treatment (Supplementary Fig. S6). Our findings collectively identified that both mutations and gene expression signatures among the treatment-naïve diagnostic tumors were associated with poor PFS following palbociclib administration. Most notably, we found that *TP53* status can be further stratified by other gene expression signatures in addition to HRD to identify individuals with poor outcomes during treatment with combination palbociclib therapy and ET among both HR^+^/HER2^−^ and HR^+^/HER^+^ breast cancers after progression on first-line ET.

### Longitudinal analyses of cfDNA from trial entry baseline, week 12, and week 24 time points identify mutations associated with poor response to palbociclib

For cfDNA profiling, 406 plasma samples were extracted across 179 patients. The baseline samples represent the time point prior to palbociclib treatment in eligible patients who failed first-line therapy and entered the trial, with collections at 12 and 24 weeks. Following postextraction and sequencing quality control, 159 baseline samples, 136 week 12 samples, and 26 week 24 samples were successfully sequenced across the 94-gene panel and are further detailed in Supplementary Fig. S7.

**Figure 3.**
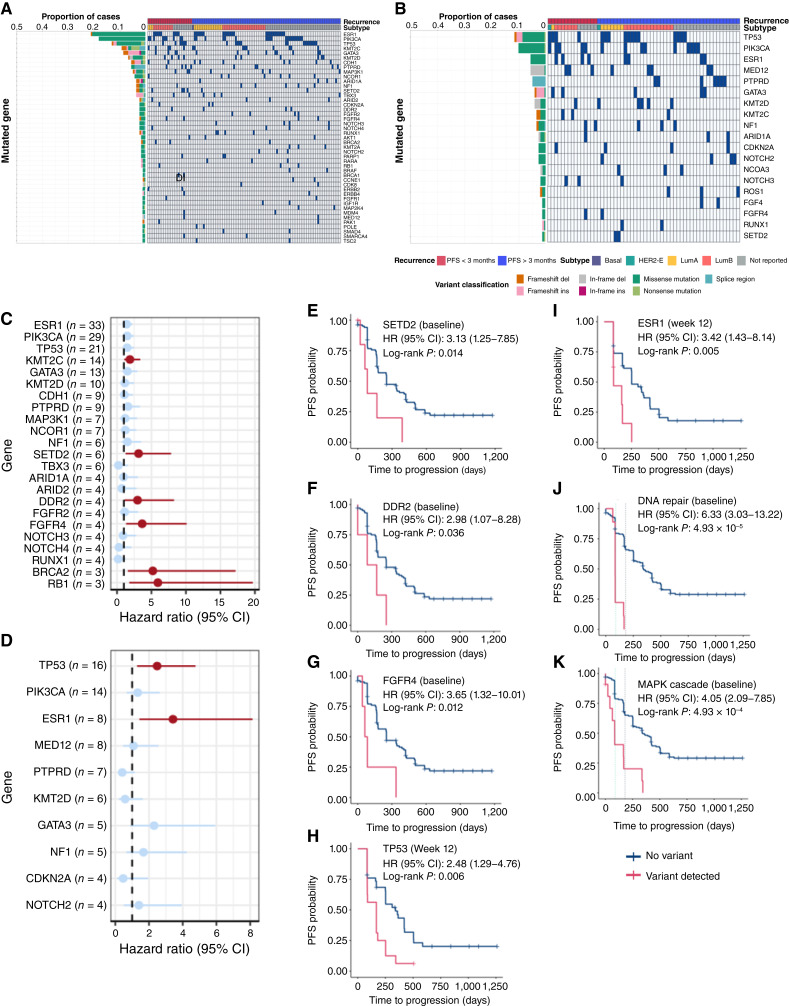
Somatic variants detected in cfDNA associated with PFS time at baseline and first follow-up at week 12. **A** and **B,** Summary of genetic variants captured at (**A**) baseline prior to palbociclib treatment and at (**B**) week 12. Blue squares indicate the detection of a somatic variant in cfDNA for individuals. Barplot colors indicate variant classification. **C** and **D,** HR associated with PFS time for genetic variants detected at (**C**) baseline and at (**D**) week 12. Red points indicate gene variants significantly associated with PFS. **E–I,** Kaplan–Meier curves of mutations significantly associated with poor PFS time at (**E–G**) baseline in (**E**) *SETD2*, (**F**) *DDR2*, and (**G**) *FGFR4* and (**H** and **I**) at week 12 in (**H**) *TP53* and (**I**) *ESR1*. **J** and **K,** Kaplan–Meier curves of mutations associated with poor PFS in groups of genes in the (**J**) DNA repair pathway and (**K**) MAPK cascade.

We observed deleterious variants in 67.9% of baseline samples (Fig. 3A), with the most frequent mutations observed in *ESR1* (21.3%), *PIK3CA* (18.2%), *TP53* (13.2%), *KMT2C* (9.4%), and *GATA3* (8.8%; Fig. 3A; Supplementary Fig. S7), which have been reported previously and are associated with resistance to AIs. We observed deleterious variants among 42.6% of cases in week 12 plasma cfDNA (Fig. 3B; Supplementary Fig. S7), including mutations in *TP53* (11.8%), followed by *PIK3CA* (10.3%), *ESR1* (5.9%), and *MED12 *(5.9%). No treatment-naïve solid tumors harbored detectable ESR1 mutations, and of the 34 participants with ESR1 cfDNA mutations at trial entry, 32 had prior exposure to AIs. Sixteen patients developed ESR1 mutations after palbociclib treatment, 12 of whom had AI treatment.

We found that variants detected among baseline samples in *SETD2* (HR = 3.13; *P* = 0.014), *DDR2* (HR = 2.98; *P* = 0.036), and *FGFR4* (HR = 3.65; *P* = 0.012) were associated with a higher risk of an event (Fig. 3C–G) on univariate Cox analysis. Comparatively, among week 12 cfDNA samples, only variants detected in *TP53* (HR = 2.48; *P* = 0.006) and *ESR1* (HR = 3.42; *P* = 0.005) were significantly associated with PFS (Fig. 3D, H, and I). Given our previous observations of the association between poor PFS and TP53 variants in pretreatment specimens, we anticipated that cases with detectable levels of TP53 at baseline or week 12 cfDNA could be a marker of palbociclib resistance. This was further highlighted by the similar frequency of cases harboring TP53 between baseline and week 12 samples (Fig. 3A and B). Conversely, we detected a decrease in the frequency of cases harboring ESR1 between baseline and week 12 (Fig. 3A and B; Supplementary Fig. S7).

Given the relatively low frequency of cases harboring mutations in most targeted genes, we further grouped genes according to biological processes to identify whether mutations within groups of genes can inform poor prognosis (Supplementary Fig. S8). Mutations in genes involved in DNA repair at the trial entry baseline sample were significantly associated with shorter PFS (HR = 6.33; log-rank *P* = 4.93 × 10^−5^) and exhibited clinical progression in most cases within 6 months (Fig. 3J). Among the genes associated with DNA repair, *NBN*, *BRCA1*, *BRCA2*, and *PALB2* were also specifically involved in double-strand break repair via homologous recombination (HR = 6.98; log-rank *P* = 1.70 × 10^−4^), concordant with previous observations of poor PFS among pretreatment solid tumors with high HRD gene expression signatures (Supplementary Fig. S8A). Additionally, mutations in genes involved with the MAPK cascade at baseline were also significantly associated with PFS (HR = 4.05; log-rank *P* = 4.93 × 10^−4^; Fig. 3K). Mutations in both week 12 samples were enriched in a high number of biological processes that included large gene sets (Supplementary Fig. S8B), which may suggest that the detectability of a tumor associated variant at follow-up time points may be more associated with PFS. No specific biological processes were significantly associated with PFS among week 24 samples.

### Relatively low tumor fraction and low short/long fragment length ratios of cfDNA at baseline are prognostic for better PFS

**Figure 4.**
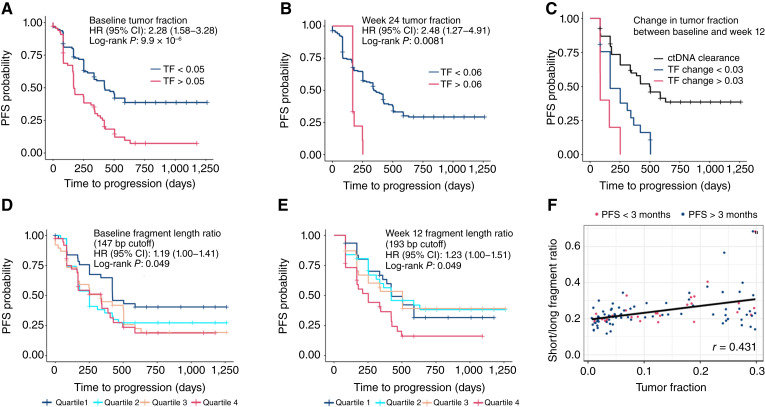
Association among tumor fraction, short/long fragment length, and PFS time. **A** and **B,** Kaplan–Meier curves of cfDNA tumor fraction cutoffs most significantly associated with poor PFS time for (**A**) baseline samples at 2% tumor fraction and (**B**) week 24 samples at 6% tumor fraction. **C,** Change in tumor fraction between baseline and week 12 associated with PFS. **D** and **E,** Kaplan–Meier curves of fragment length ratio cutoffs (**D**) at baseline and (**E**) week 12. **F,** Correlation between tumor fraction and short/long fragment length ratio at baseline.

We estimated the tumor fraction among cases by taking the highest nonsilent somatic mutation VAF per sample to inform PFS. For each time point, the optimal tumor fraction cutoff prognostic of PFS was assessed by performing a univariate Cox regression analysis between samples above and below specific tumor fraction thresholds. Among the baseline samples, any tumor fraction higher than 1% was significantly associated with shorter PFS (Supplementary Fig. S9A); however, a cutoff higher than 5% yielded the most significant association (HR = 2.28; log-rank *P* = 9.9 × 10^−6^), with evidence of tumor progression in most of these cases within 2 years (Fig. 4A). A tumor fraction threshold of 2% for week 12 samples was significantly associated with PFS (HR = 1.48; log-rank *P* = 0.0462; Supplementary Fig. S9A), whereas at week 12, any tumor fraction greater than 1% was significantly associated with shorter PFS, most significantly at a tumor fraction threshold of 6% (HR = 2.48; log-rank *P* = 0.0081), with all cases greater than this threshold developing progression within a year (Fig. 4B).

We compared whether a change in tumor fraction between the baseline sample prior to palbociclib and at week 12 on treatment was associated with outcomes. Cases with detectable levels of cfDNA tumor mutations at both baseline and week 12 exhibited significantly worse PFS, regardless of the directionality of change in tumor fraction, whereas cases that no longer had detectable tumor variants at week 12 were associated with improved PFS (Fig. 4C). When taking into consideration the changes in cfDNA tumor fraction among cases with detectable variants at week 12, we observed that cases with a 3% or higher increase in cfDNA VAF were most significantly associated with PFS and clinical progression within 8 months (Fig. 4C; Supplementary Fig. S9B).

Multiple studies have demonstrated that shorter cfDNA fragments are predictive of early progression and minimal residual disease by computing the ratio of short/long fragments. Our observed cfDNA fragment length distributions across patient samples were concordant with previously reported distributions (23, 24), peaking at 167 bp with 10 bp periodicity below 150 bp (Supplementary Fig. S9C). Fragment length cutoffs of 147 and 193 bp at baseline and week 12, respectively, demonstrated significant associations with PFS after iterative assessment of different short fragment thresholds (Supplementary Fig. S9D). Patients with the lowest short/long fragment ratios (quartile 1) at baseline experienced better PFS (HR = 1.19; log-rank *P* = 0.049; Fig. 4D), whereas at week 12, patients with the highest short/long fragment ratios (quartile 4) at the 193 bp cut point experienced significantly worse PFS (HR = 1.23; log-rank *P* = 0.049; Fig. 4E). Although fragment length ratios at baseline cutoff were positively correlated with tumor fraction (Fig. 4F), we found that tumor fraction was comparatively more prognostic of PFS.

## Discussion

Determination of the optimal dosing strategy for targeted agents is particularly challenging as target inhibition may not have a direct relationship to toxicity, and a plateau in target inhibition may be achieved at dose levels lower than the maximum tolerated dose. The exploration of alternate dose schedules of approved targeted therapies is of great interest to the oncology community due to potential gains in efficacy, tolerability, ease of administration, and potential cost savings (27, 28). Although the *in silico* modeling suggested that CDD palbociclib may be associated with less neutropenia and greater antitumoral activity than SDS palbociclib, we instead found greater rates of grade 3 and 4 neutropenia (without increased rates of febrile neutropenia), leading to more dose reductions in the CDD schedule and no consistent signal of improved efficacy. This may be explained by the difference in protocol-mandated dose modification schedules, especially for uncomplicated grade 3 neutropenia, which is commonly observed with CDK 4/6 inhibitor therapy, leading to differences in drug exposure favoring the intermittent SDS compared with the CDD arms, including median daily dose, cumulative dose, and dose intensity.

We speculate that the use of a less aggressive dose modification algorithm in the CDD arm may have led to more drug exposure and greater efficacy compared with the SDS arm. Ultimately, the tolerability of the 100 mg daily dose schedule is supported by the lack of significant differences in QOL outcomes compared with the SDS. We note that ribociclib and abemaciclib are approved CDK4/6 inhibitors for the treatment of early and advanced breast cancer and have also been shown to be effective at a lower dose than the approved starting dose (29–31). Furthermore, abemaciclib is administered on a continuous dosing schedule, and similar outcomes have been demonstrated in the adjuvant settings for lower drug exposure rates compared with the approved dose schedule (30).

The extensive correlative studies sought to identify prognostic biomarkers. Only mutations in *TP53*, *CDH1*, and *IRS2* were significantly associated with shorter PFS in the treatment-naïve diagnostic tumors. Park and colleagues (26) reported that mutations of TP53 at baseline were associated with shorter PFS in a cohort of patients treated with palbociclib plus ET in matched pretreatment tumor biopsies, on-treatment tissues, and progression tissues and also integrated HRD features to demonstrate shorter PFS. MA.38 solid tissue RNA profiling demonstrated that a low HRD gene expression score with no TP53 mutations resulted in a better PFS than tumors with high HRD expression scores and TP53 mutations. TP53 mutations were frequently identified among the Luminal B cancers, consistent with the characteristically higher proliferation associated with the subtype (32, 33). In addition, cases identified as non-luminal (basal-like or HER2-E) were also characterized by TP53 mutations and a high genomic risk score. Cejalvo and colleagues (34) reported that the rate of PAM50 subtype conversion was most frequent in luminal A (55.3%) and luminal B (30.0%) tumors, ultimately changing at metastasis to luminal B (in 40.2% of luminal A) and HER2-E (in 14.3% of luminal A and B). Basal-like tumors were found not to convert at metastasis, and only 23% of HER2-E converted. Although there were no samples available following first-line treatment failure to observe the extent of conversion, it is likely that a proportion of these advanced patients did exhibit molecular features like more aggressive luminal B or non-luminal subtypes, as suggested by the mutational spectrum in the ctDNA profiles, particularly TP53 mutations, captured at treatment failure and before palbociclib treatment.

Enrichment of known pathways/genes associated with HR positivity was observed, including ER signaling, strong FOXA1 and TGFβ expression, and enrichment of gene expression associated with the antigen-presenting machinery, consistent with the generally immune-cold features of luminal cancers (35). These findings highlight the contributing role of the tumor microenvironment, with patients possessing higher macrophage, stemness, and stromal gene signatures also experiencing poorer PFS, features of more aggressive breast cancer (36, 37). The TIS (19) was shown in our data to be prognostic in the context of *TP53* and HRD stratification. High TIS scores, mutations in *TP53*, and features of HRD are common characteristics of more aggressive luminal breast cancer phenotypes as well as basal-like/triple negative breast cancers (37), suggesting that endocrine-based therapies and CDK4/6 inhibition may not be adequate and that other targeted therapies, such as PARP inhibitors or immmunotherapies might be better matched to these tumor biologies despite their positive HR status. To that end, findings from the International Immuno-Oncology Biomarker Working Group (38) and spatial biology (39, 40) studies are highlighting the impact of the tumor microenvironment on breast cancer prognosis and response to treatment.

In the baseline plasma at trial entry, cfDNA mutations in *ESR1* were most frequently identified in patients (21.3%), followed by mutations in *PIK3CA* (18.2%), *TP53* (13.2%), *KMT2C* (9.4%), and *GATA3* (8.8%), consistent with other studies (41–46). ESR1 mutations have been previously observed to be a common cause of acquired resistance to ET and are well documented as a genomic feature of progressive or metastatic disease on adjuvant antihormone therapies. No ESR1 mutations were detected in the matching treatment-naïve diagnostic solid tissues, suggesting the acquisition of ESR1 mutations and expansion of treatment-resistant subclones in response to prior ET (42, 44, 47, 48). Decreasing numbers of patients with ESR1 mutations between baseline and week 12 are suggestive of palbociclib efficacy in a subset of patients (49). Cases harboring detectable ESR1 mutations at week 12 were significantly associated with poor PFS, suggesting that the poor response to palbociclib results in sustained detection of tumor-associated variants in cfDNA. This is reflective of tumor resistance rather than ESR1 conferring resistance to the treatment, as previously documented by Lloyd and colleagues (50), who reported that ESR1 variants are not associated with pan-CDK4/6 inhibitor resistance.

Although a good prognostic marker in the early disease setting (51, 52), *PIK3CA* mutations in the advanced setting are associated with poorer outcomes. Neither PIK3CA mutations in the treatment-naïve diagnostic tissues nor those in trial entry baseline samples were significant for associations with PFS in this cohort, in keeping with other CDK4/6 studies (44, 53). Mirroring the results in the solid tissues, the presence of circulating *TP53* mutations at baseline and week 12 were markers of poor prognosis, similar to results obtained from MONALEESA-2 (41), PALOMA-3 (42, 43), and others (42, 44–46). However, TP53 mutations are also associated with clonal hematopoiesis (CH; ref. 54), and a limitation of this study was the absence of analysis for the matching buffy coat samples; thus, we cannot exclude the impact of CH on these findings. Despite this, our mutational rate for TP53 was 13.2%, comparable with that seen for PALOMA-3 (15.4%; refs. 42, 43). Most recently, Fairchild and colleagues (55) reported that the proportion of TP53 variants due to CH in matched blood and tissue is low among HR+ breast cancer at approximately 0.1%.

Pathway-centric analyses using the genes in the cfDNA panel showed that aberrations among DNA repair genes at baseline were associated with shorter PFS (HR = 6.33; *P* = 4.93 × 10^−5^), recapitulating transcriptional observations in the diagnostic treatment-naïve solid tissues, suggesting that first-line treatment of et alone may be insufficient for those affected by DNA repair pathway aberrations. The prominent findings of both genomic and transcriptomic perturbations in HRD and cell-cycle pathways in both primary tissues and cfDNA obtained at treatment failure suggest that a subset of patients might benefit from PARP inhibitors (56). Similarly, mutations in genes in the MAPK cascade were also significantly associated with poor PFS (HR = 4.05; *P* = 4.93 × 10^−4^), and we observed mutations in this pathway prior to palbociclib treatment, suggesting that MAPK-reliant palbociclib-resistant clones could potentially be detected from plasma cfDNA prior to treatment. Results from the FELINE trial (57) showed that patients treated with both ribociclib and letrozole in the early setting had upregulation of estrogen-independent proliferation through various pathways, including JNK and ERK, which are major components of the MAPK network, and this could be a biomarker for CDK4/6 resistance.

Using measures of cfDNA dynamics, a significant correlation with PFS was identified among baseline cfDNA samples with a tumor fraction greater than 5% (HR = 2.28; *P* = 9.9 × 10^−8^), with a progression event within 2 years. At week 12 of treatment, a tumor fraction threshold of 2% was significantly associated with PFS (HR = 1.48; *P* = 0.0462), whereas at week 24, a threshold of 1% was significant for progression within a year (HR = 2.48; *P* = 0.0081). Similarly, we explored the change in tumor fraction between the pretreatment cfDNA sample and the week 12 time point, which showed that ctDNA clearance, indicated by the absence of any tumor-associated variants, was significantly associated with better PFS. In fact, all cases with detectable cfDNA by 12 weeks experienced a progression event within 1.5 years, whereas cases harboring a 3% or greater increase in VAF at week 12 experienced a progression event within 8 months, which is consistent with findings in other studies (58). Finally, several studies have now investigated the cfDNA fragment size as a measure of residual disease (23, 24). In our cohort, patients with the lowest short/long ratio at baseline had an improved PFS (HR = 1.19; *P* = 0.049), in contrast to ratio changes at week 12, in which higher short/long fragment ratios had a worse PFS (HR = 1.23; *P* = 0.049). Collectively, our findings highlight the utility of cfDNA biomarkers for minimal residual disease and that the detection of ctDNA fragments can be utilized as prognostic indicators of PFS to inform treatment efficacy. Although the targeted panel utilized was limited in its ability to identify all cases with poor PFS, as more data from liquid biopsies in the larger targeted panels and whole-genome space become more readily available, the ability to identify new biomarkers and establish appropriate thresholds and cut points will permit comprehensive clinical utility studies.

In summary, our results indicate that 100 mg CDD of palbociclib as administered in our trial is tolerable but not associated with improved efficacy signals compared with the 125 mg SDS. Correlative studies revealed that TP53 mutations and mutational and transcriptional profiles at diagnosis among genes in the HRD and DNA repair pathways were prognostic for poor PFS. We also demonstrated that changes in tumor fraction were associated with PFS, as were changes in the fragment size ratios, and that such information can be derived from a small, targeted panel, offering the potential for a practical and rapid method for measuring disease progression in patients. Finally, a pathway-driven approach combined with cfDNA dynamics may provide a better understanding of disease progression, tumor burden, and opportunities to inform a personalized treatment approach for existing and future novel therapeutic interventions.

## Supplementary Material

Supplementary File 1 Supplementary File 1 - cfDNA panel description

Supplementary Figure S1 Figure S1. Solid tissue and liquid biopsy MA38 correlative sciences cohort

Supplementary Figure S2 Figure S2. Summary of genomic solid tissue results.

Supplementary Figure S3 Figure S3. Summary of gene signature results from the NanoString Breast Cancer 360 (BC360) Panel in treatment-naive solid tissues at diagnosis stratified by progression status.

Supplementary Figure S4 Figure S4. Top differentially expressed genes associated with PFS in treatment-naive solid tissues at diagnosis.

Supplementary Figure S5 Figure S5. Top gene expression signatures associated with PFS in treatment-naive solid tissues at diagnosis.

Supplementary Figure S6 Supplementary Figure S6. Prognostic transcriptional signatures stratified by TP53 mutation and BC360 HRD expression status in solid tissues.

Supplementary Figure S7 Figure S7. Summary of top mutated genes in solid tissue and longitudinal liquid biopsy samples.

Supplementary Figure S8 Figure S8. Summary of biological processes associated with PFS in baseline and W12 cfDNA plasma based on mutational profiles. (A-B) Forest plots summarize HR of mutations in genes among biological processes significantly associated with PFS among (A) baseline and (B) W12 samples.

Supplementary Figure S9 Figure S9. Association between tumour-fraction, short to long fragment length ratios and progression-free survival time. A) Hazard ratio between individuals above and below each tumour fraction cut-off at baseline (green), W12 (red) and W24 (yellow). Filled in points indicate significant hazard ratios. B) Hazard ratios of tumour fraction change between baseline and W12 cfDNA associated with progression-free survival at various cut-offs. Filled in points indicate significant hazard ratios. C) Cell-free DNA fragment length distribution between individuals with shorter and longer progression free survival time of three months. D) Hazard ratio of short to long fragment length ratio on progression free survival time using various cut-offs to divide short and long. Colours indicate cfDNA sampling time at baseline (green), W12 (red) and at W24 (yellow). Filled in points are significant log-rank p-values fragment length cut-offs.

Supplementary Table 1 Supplementary Table 1. Breast Cancer 360 Gene Signatures and Description

Supplementary Table 2 Supplementary Table 2. Palbociclib Drug Exposure

Supplementary Table 3 Supplementary Table 3. Incidence Adverse Events (greater than 20% of patients for non-hematological AEs and greater than 10% for haematological/ chemistry AEs)

## Data Availability

The data generated in this study are available from the corresponding author upon request. Data were generated by the authors and included in the article.
