# Supplementary material for: Continuous versus Standard Palbociclib Treatment and Molecular Profiling of Solid Tissues and Liquid Biopsies in the CCTG MA.38 Trial in Advanced Breast Cancer
Source: Cancer Res Commun. 2025 Nov 13;5(11):1998–2011. doi: 10.1158/2767-9764.CRC-25-0346 (PMC12613153; doi:10.1158/2767-9764.CRC-25-0346)
Supplement: Supplementary Figure S1 — Figure S1. Solid tissue and liquid biopsy MA38 correlative sciences cohort [file crc-25-0346_supplementary_figure_s1_suppsf1.pptx]

## Slide 1
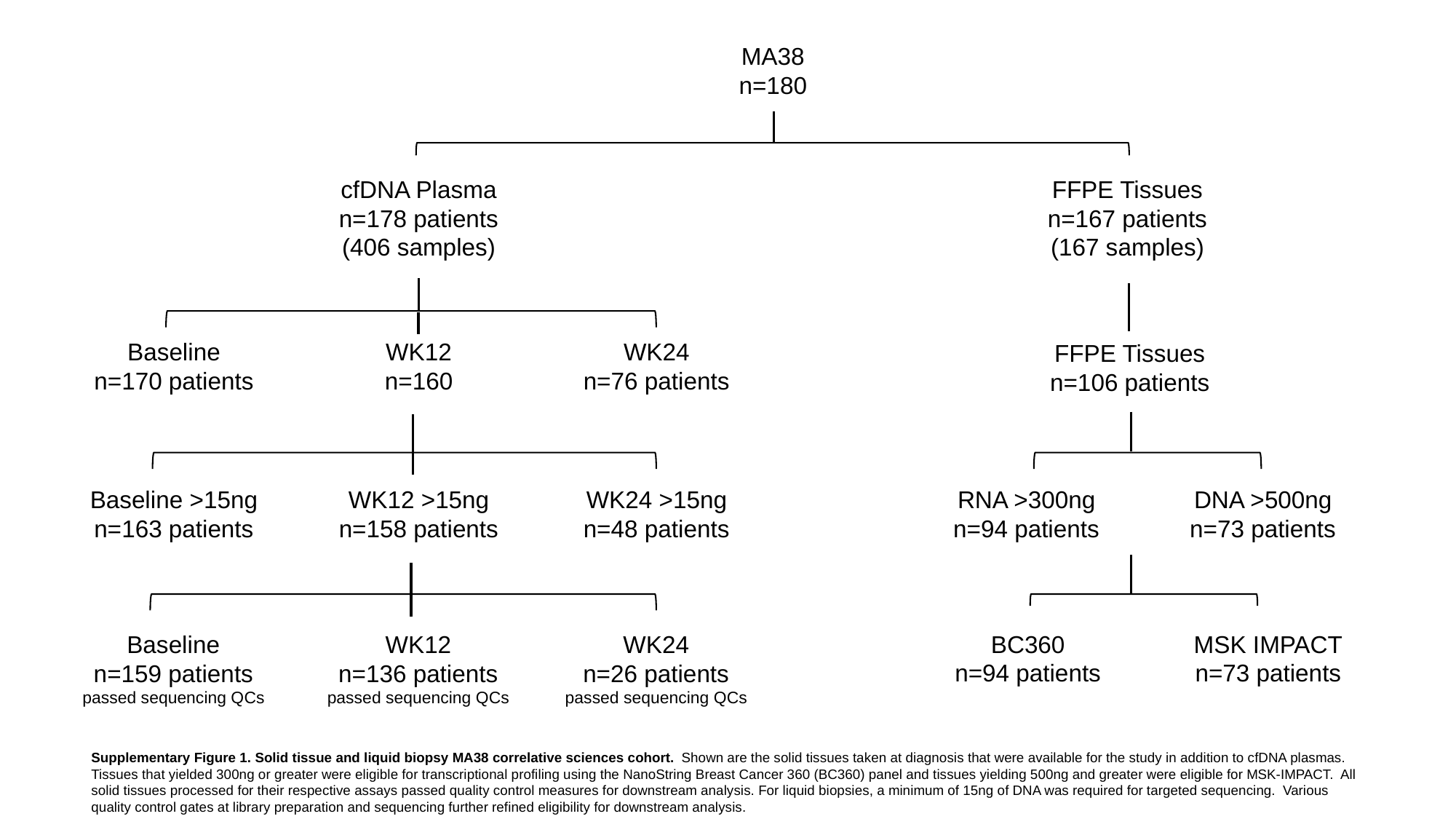

MA38
n=180
cfDNA Plasma
n=178 patients
(406 samples)
FFPE Tissues
n=167 patients
(167 samples)
WK24
n=76 patients
Baseline
n=170 patients
WK12
n=160
FFPE Tissues
n=106 patients
WK24 >15ng
n=48 patients
Baseline >15ng
n=163 patients
WK12 >15ng
n=158 patients
DNA >500ng
n=73 patients
RNA >300ng
n=94 patients
WK24
n=26 patients
passed sequencing QCs
Baseline
n=159 patients
passed sequencing QCs
WK12
n=136 patients
passed sequencing QCs
MSK IMPACT
n=73 patients
BC360
n=94 patients
Supplementary Figure 1. Solid tissue and liquid biopsy MA38 correlative sciences cohort. Shown are the solid tissues taken at diagnosis that were available for the study in addition to cfDNA plasmas. Tissues that yielded 300ng or greater were eligible for transcriptional profiling using the NanoString Breast Cancer 360 (BC360) panel and tissues yielding 500ng and greater were eligible for MSK-IMPACT. All solid tissues processed for their respective assays passed quality control measures for downstream analysis. For liquid biopsies, a minimum of 15ng of DNA was required for targeted sequencing. Various quality control gates at library preparation and sequencing further refined eligibility for downstream analysis.
