# Supplementary material for: Continuous versus Standard Palbociclib Treatment and Molecular Profiling of Solid Tissues and Liquid Biopsies in the CCTG MA.38 Trial in Advanced Breast Cancer
Source: Cancer Res Commun. 2025 Nov 13;5(11):1998–2011. doi: 10.1158/2767-9764.CRC-25-0346 (PMC12613153; doi:10.1158/2767-9764.CRC-25-0346)
Supplement: Supplementary Figure S2 — Figure S2. Summary of genomic solid tissue results. [file crc-25-0346_supplementary_figure_s2_suppsf2.pptx]

## Slide 1
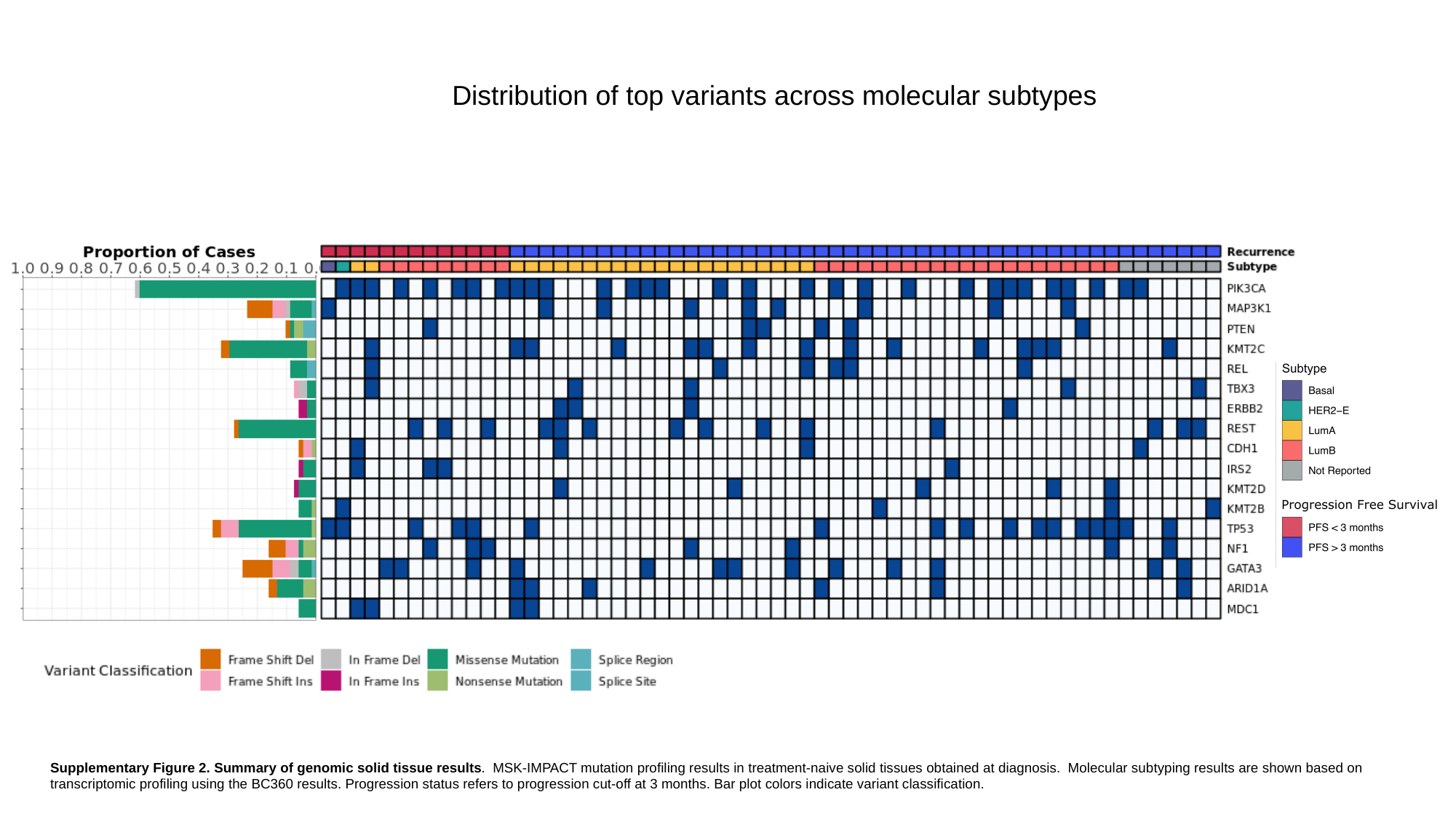

Distribution of top variants across molecular subtypes
Supplementary Figure 2. Summary of genomic solid tissue results. MSK-IMPACT mutation profiling results in treatment-naive solid tissues obtained at diagnosis. Molecular subtyping results are shown based on transcriptomic profiling using the BC360 results. Progression status refers to progression cut-off at 3 months. Bar plot colors indicate variant classification.
