# Supplementary material for: Continuous versus Standard Palbociclib Treatment and Molecular Profiling of Solid Tissues and Liquid Biopsies in the CCTG MA.38 Trial in Advanced Breast Cancer
Source: Cancer Res Commun. 2025 Nov 13;5(11):1998–2011. doi: 10.1158/2767-9764.CRC-25-0346 (PMC12613153; doi:10.1158/2767-9764.CRC-25-0346)
Supplement: Supplementary Figure S3 — Figure S3. Summary of gene signature results from the NanoString Breast Cancer 360 (BC360) Panel in treatment-naive solid tissues at diagnosis stratified by progression status. [file crc-25-0346_supplementary_figure_s3_suppsf3.pptx]

## Slide 1
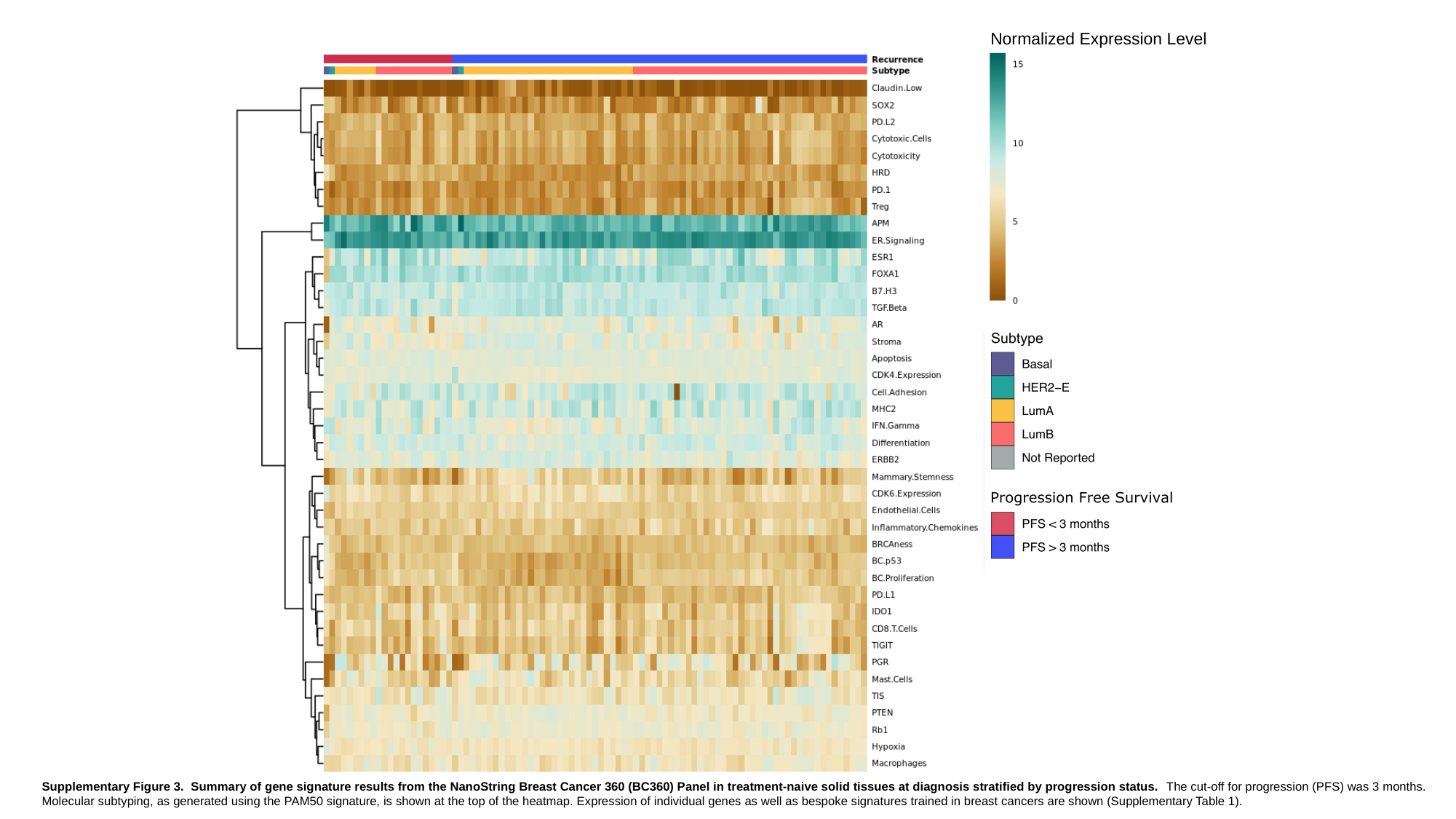

Normalized Expression Level
Supplementary Figure 3. Summary of gene signature results from the NanoString Breast Cancer 360 (BC360) Panel in treatment-naive solid tissues at diagnosis stratified by progression status. The cut-off for progression (PFS) was 3 months. Molecular subtyping, as generated using the PAM50 signature, is shown at the top of the heatmap. Expression of individual genes as well as bespoke signatures trained in breast cancers are shown (Supplementary Table 1).
