# Supplementary material for: Continuous versus Standard Palbociclib Treatment and Molecular Profiling of Solid Tissues and Liquid Biopsies in the CCTG MA.38 Trial in Advanced Breast Cancer
Source: Cancer Res Commun. 2025 Nov 13;5(11):1998–2011. doi: 10.1158/2767-9764.CRC-25-0346 (PMC12613153; doi:10.1158/2767-9764.CRC-25-0346)
Supplement: Supplementary Figure S4 — Figure S4. Top differentially expressed genes associated with PFS in treatment-naive solid tissues at diagnosis. [file crc-25-0346_supplementary_figure_s4_suppsf4.pptx]

## Slide 1
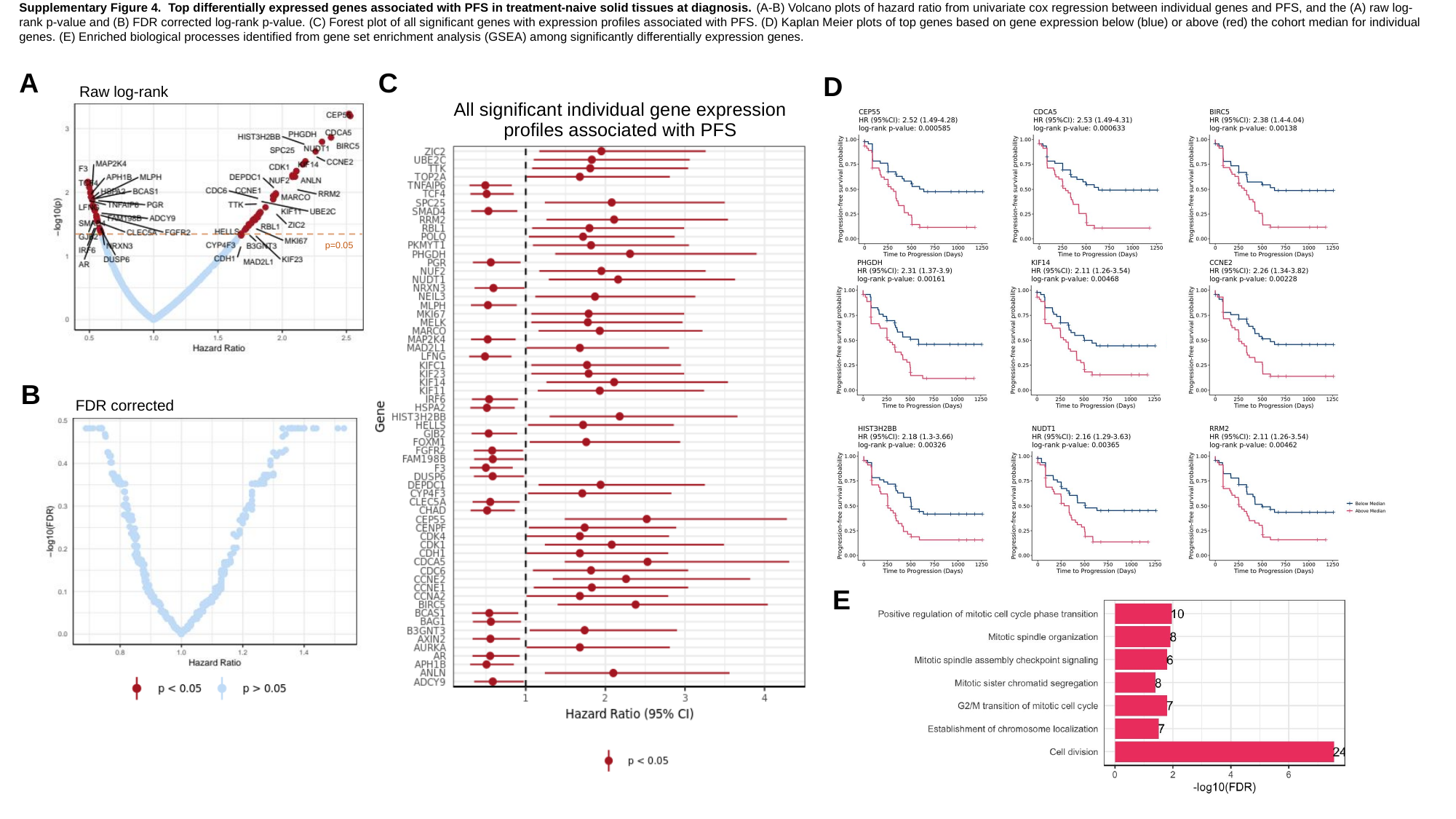

Supplementary Figure 4. Top differentially expressed genes associated with PFS in treatment-naive solid tissues at diagnosis. (A-B) Volcano plots of hazard ratio from univariate cox regression between individual genes and PFS, and the (A) raw log-rank p-value and (B) FDR corrected log-rank p-value. (C) Forest plot of all significant genes with expression profiles associated with PFS. (D) Kaplan Meier plots of top genes based on gene expression below (blue) or above (red) the cohort median for individual genes. (E) Enriched biological processes identified from gene set enrichment analysis (GSEA) among significantly differentially expression genes.
All significant individual gene expression profiles associated with PFS
A
C
D
Raw log-rank
p=0.05
B
FDR corrected
E
