# Supplementary material for: Continuous versus Standard Palbociclib Treatment and Molecular Profiling of Solid Tissues and Liquid Biopsies in the CCTG MA.38 Trial in Advanced Breast Cancer
Source: Cancer Res Commun. 2025 Nov 13;5(11):1998–2011. doi: 10.1158/2767-9764.CRC-25-0346 (PMC12613153; doi:10.1158/2767-9764.CRC-25-0346)
Supplement: Supplementary Figure S5 — Figure S5. Top gene expression signatures associated with PFS in treatment-naive solid tissues at diagnosis. [file crc-25-0346_supplementary_figure_s5_suppsf5.pptx]

## Slide 1
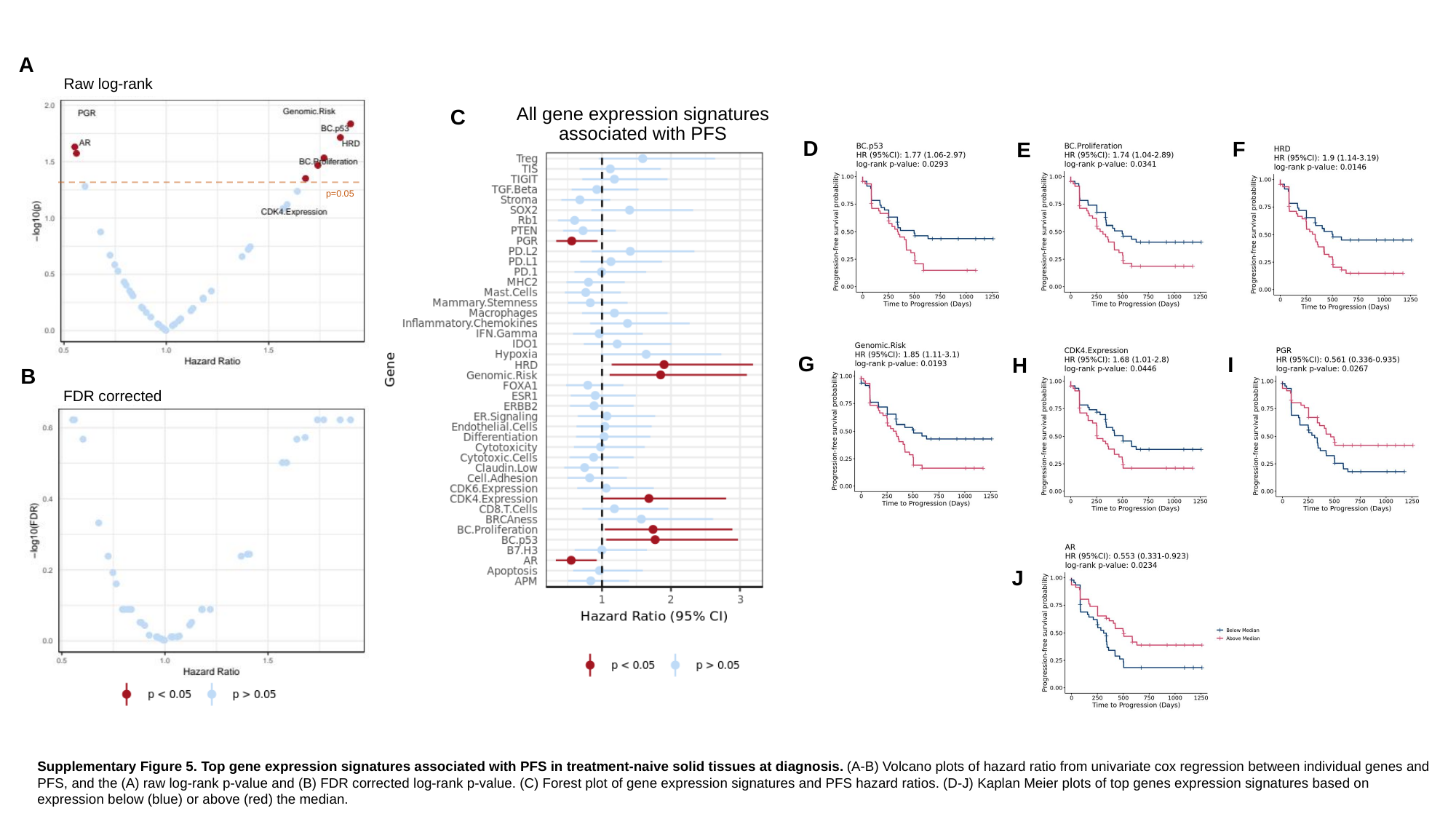

A
All gene expression signatures associated with PFS
Raw log-rank
C
D
F
E
p=0.05
G
I
H
B
FDR corrected
J
Supplementary Figure 5. Top gene expression signatures associated with PFS in treatment-naive solid tissues at diagnosis. (A-B) Volcano plots of hazard ratio from univariate cox regression between individual genes and PFS, and the (A) raw log-rank p-value and (B) FDR corrected log-rank p-value. (C) Forest plot of gene expression signatures and PFS hazard ratios. (D-J) Kaplan Meier plots of top genes expression signatures based on expression below (blue) or above (red) the median.
