# Supplementary material for: Continuous versus Standard Palbociclib Treatment and Molecular Profiling of Solid Tissues and Liquid Biopsies in the CCTG MA.38 Trial in Advanced Breast Cancer
Source: Cancer Res Commun. 2025 Nov 13;5(11):1998–2011. doi: 10.1158/2767-9764.CRC-25-0346 (PMC12613153; doi:10.1158/2767-9764.CRC-25-0346)
Supplement: Supplementary Figure S6 — Prognostic transcriptional signatures stratified by TP53 mutation and BC360 HRD expression status in solid tissues. [file crc-25-0346_supplementary_figure_s6_suppsf6.pptx]

## Slide 1
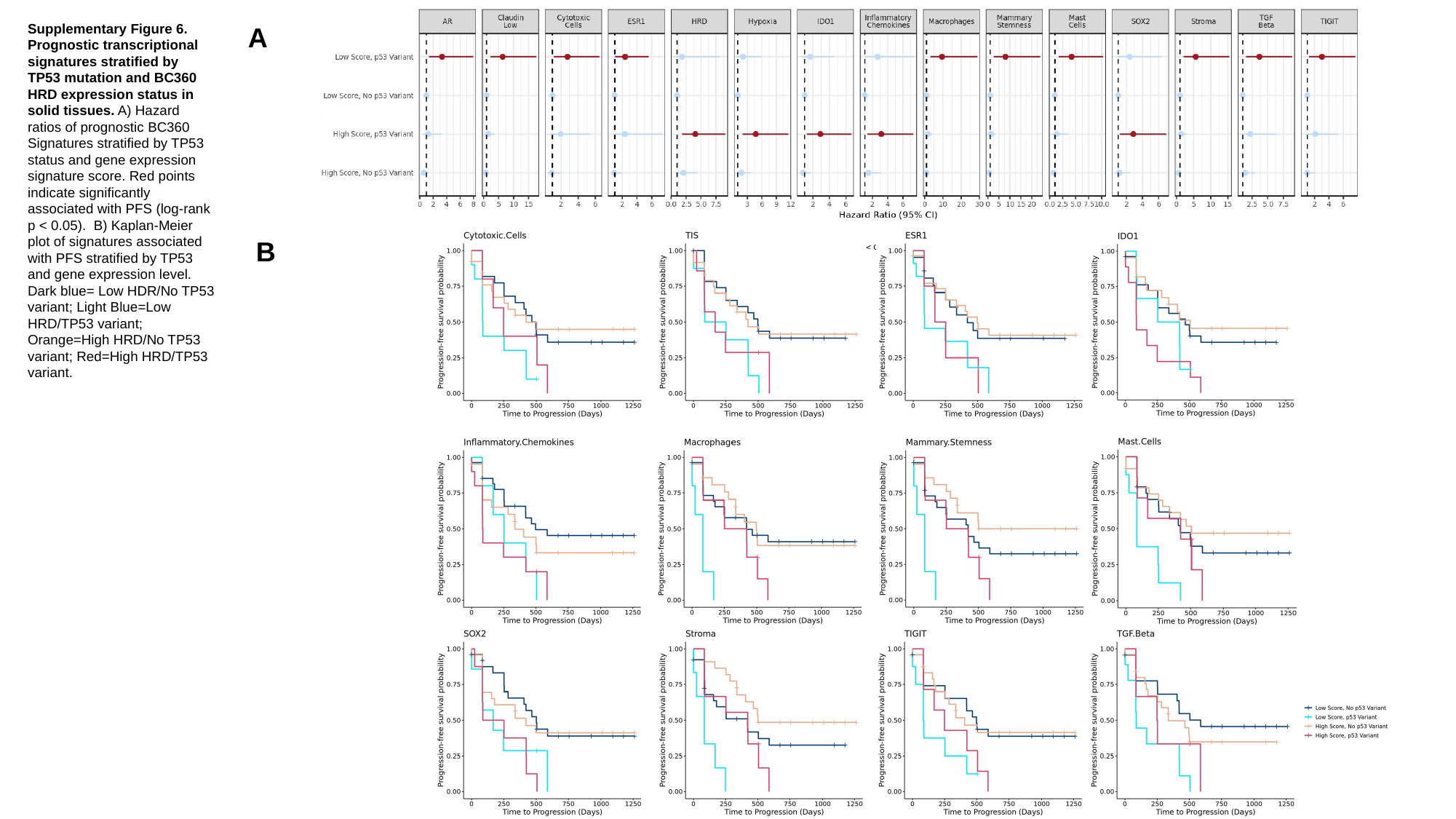

Supplementary Figure 6. Prognostic transcriptional signatures stratified by TP53 mutation and BC360 HRD expression status in solid tissues. A) Hazard ratios of prognostic BC360 Signatures stratified by TP53 status and gene expression signature score. Red points indicate significantly associated with PFS (log-rank p < 0.05). B) Kaplan-Meier plot of signatures associated with PFS stratified by TP53 and gene expression level. Dark blue= Low HDR/No TP53 variant; Light Blue=Low HRD/TP53 variant; Orange=High HRD/No TP53 variant; Red=High HRD/TP53 variant.
A
B
