# Supplementary material for: Continuous versus Standard Palbociclib Treatment and Molecular Profiling of Solid Tissues and Liquid Biopsies in the CCTG MA.38 Trial in Advanced Breast Cancer
Source: Cancer Res Commun. 2025 Nov 13;5(11):1998–2011. doi: 10.1158/2767-9764.CRC-25-0346 (PMC12613153; doi:10.1158/2767-9764.CRC-25-0346)
Supplement: Supplementary Figure S7 — Figure S7. Summary of top mutated genes in solid tissue and longitudinal liquid biopsy samples. [file crc-25-0346_supplementary_figure_s7_suppsf7.pptx]

## Slide 1
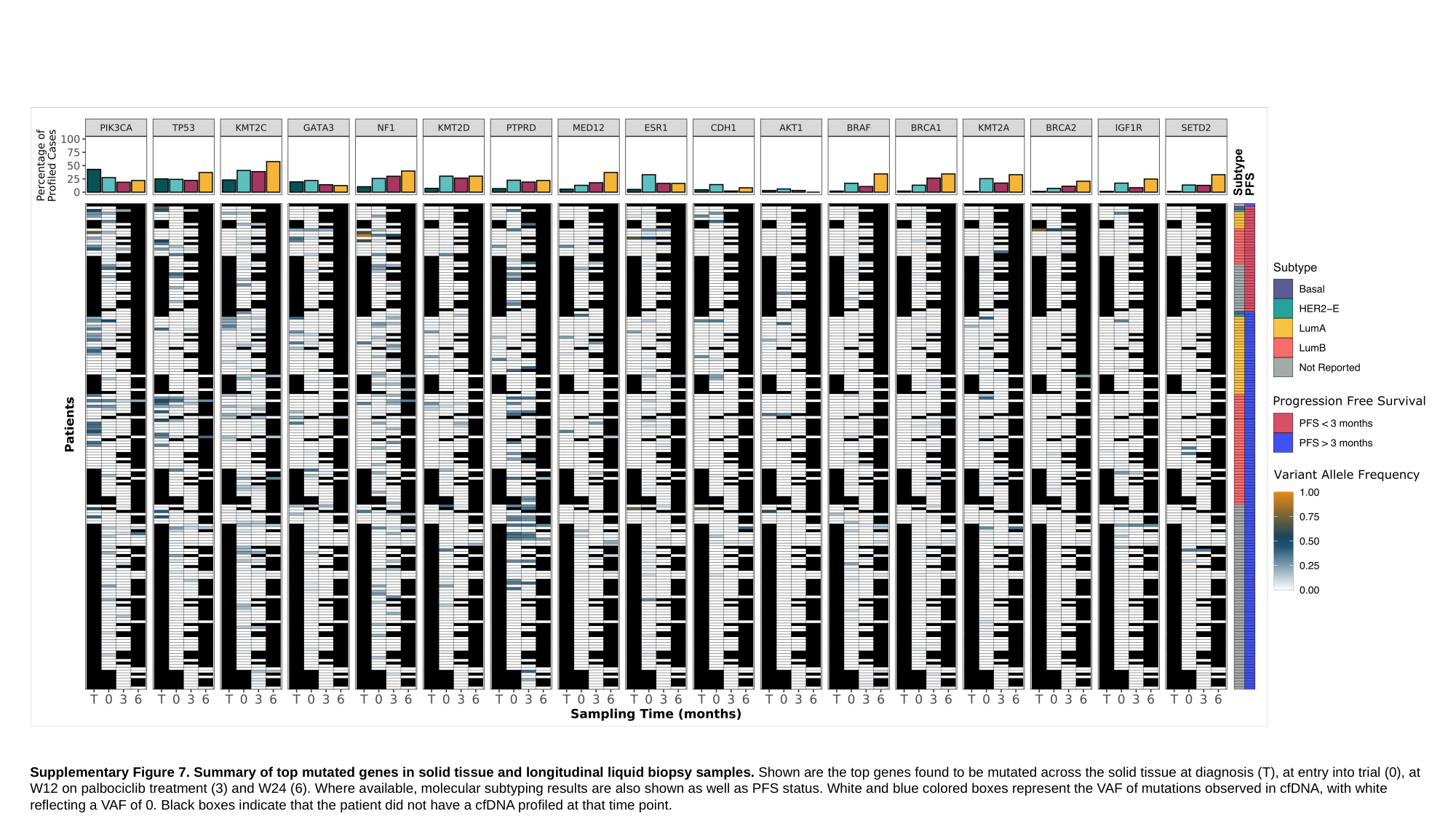

Supplementary Figure 7. Summary of top mutated genes in solid tissue and longitudinal liquid biopsy samples. Shown are the top genes found to be mutated across the solid tissue at diagnosis (T), at entry into trial (0), at W12 on palbociclib treatment (3) and W24 (6). Where available, molecular subtyping results are also shown as well as PFS status. White and blue colored boxes represent the VAF of mutations observed in cfDNA, with white reflecting a VAF of 0. Black boxes indicate that the patient did not have a cfDNA profiled at that time point.
