# Supplementary material for: Continuous versus Standard Palbociclib Treatment and Molecular Profiling of Solid Tissues and Liquid Biopsies in the CCTG MA.38 Trial in Advanced Breast Cancer
Source: Cancer Res Commun. 2025 Nov 13;5(11):1998–2011. doi: 10.1158/2767-9764.CRC-25-0346 (PMC12613153; doi:10.1158/2767-9764.CRC-25-0346)
Supplement: Supplementary Figure S8 — Figure S8. Summary of biological processes associated with PFS in baseline and W12 cfDNA plasma based on mutational profiles. (A-B) Forest plots summarize HR of mutations in genes among biological processes significantly associated with PFS among (A) baseline and (B) W12 samples. [file crc-25-0346_supplementary_figure_s8_suppsf8.pptx]

## Slide 1
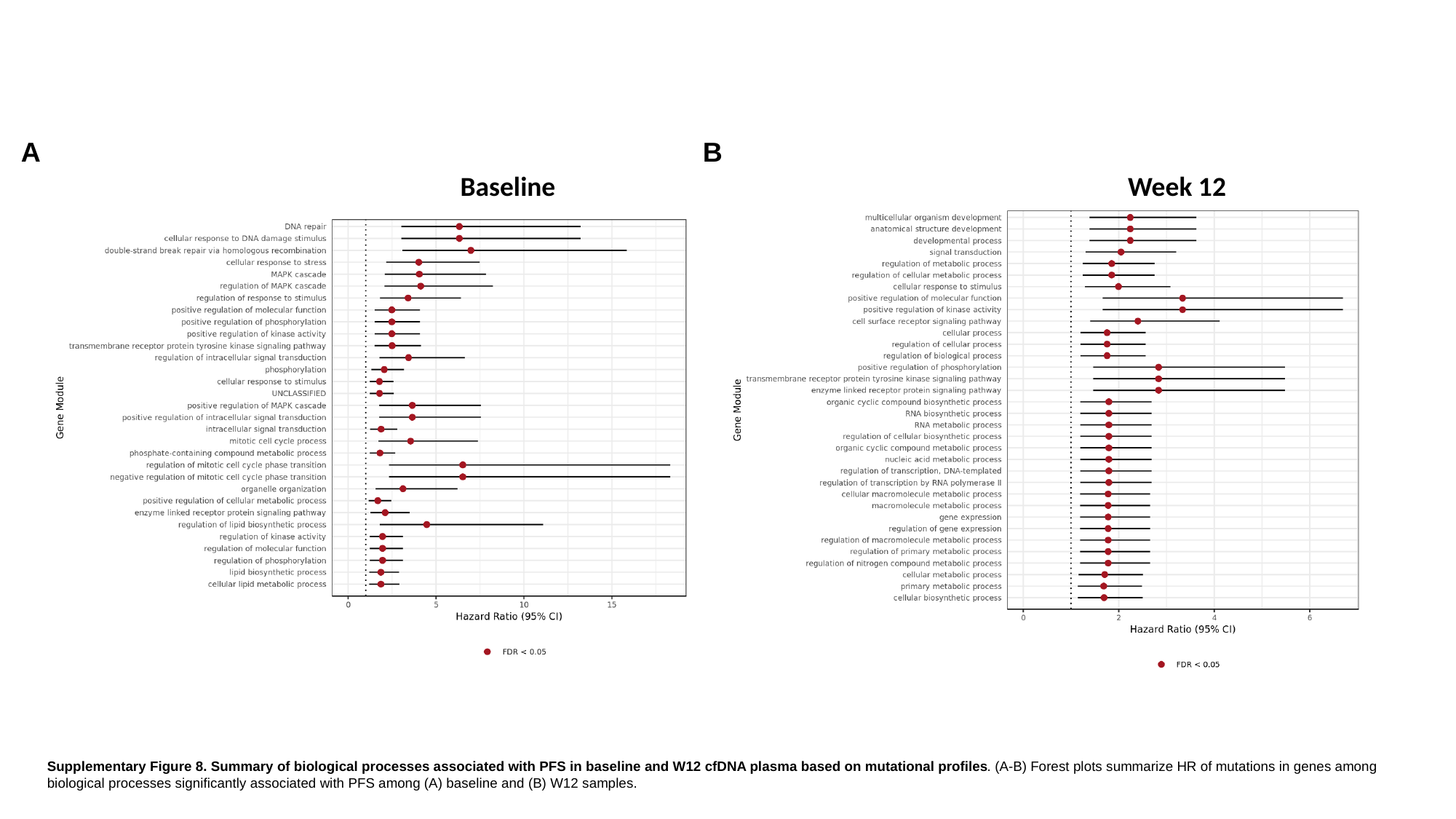

A
B
Baseline
Week 12
Supplementary Figure 8. Summary of biological processes associated with PFS in baseline and W12 cfDNA plasma based on mutational profiles. (A-B) Forest plots summarize HR of mutations in genes among biological processes significantly associated with PFS among (A) baseline and (B) W12 samples.
