# Supplementary material for: Continuous versus Standard Palbociclib Treatment and Molecular Profiling of Solid Tissues and Liquid Biopsies in the CCTG MA.38 Trial in Advanced Breast Cancer
Source: Cancer Res Commun. 2025 Nov 13;5(11):1998–2011. doi: 10.1158/2767-9764.CRC-25-0346 (PMC12613153; doi:10.1158/2767-9764.CRC-25-0346)
Supplement: Supplementary Figure S9 — Figure S9. Association between tumour-fraction, short to long fragment length ratios and progression-free survival time. A) Hazard ratio between individuals above and below each tumour fraction cut-off at baseline (green), W12 (red) and W24 (yellow). Filled in points indicate significant hazard ratios. B) Hazard ratios of tumour fraction change between baseline and W12 cfDNA associated with progression-free survival at various cut-offs. Filled in points indicate significant hazard ratios. C) Cell-free DNA fragment length distribution between individuals with shorter and longer progression free survival time of three months. D) Hazard ratio of short to long fragment length ratio on progression free survival time using various cut-offs to divide short and long. Colours indicate cfDNA sampling time at baseline (green), W12 (red) and at W24 (yellow). Filled in points are significant log-rank p-values fragment length cut-offs. [file crc-25-0346_supplementary_figure_s9_suppsf9.pptx]

## Slide 1
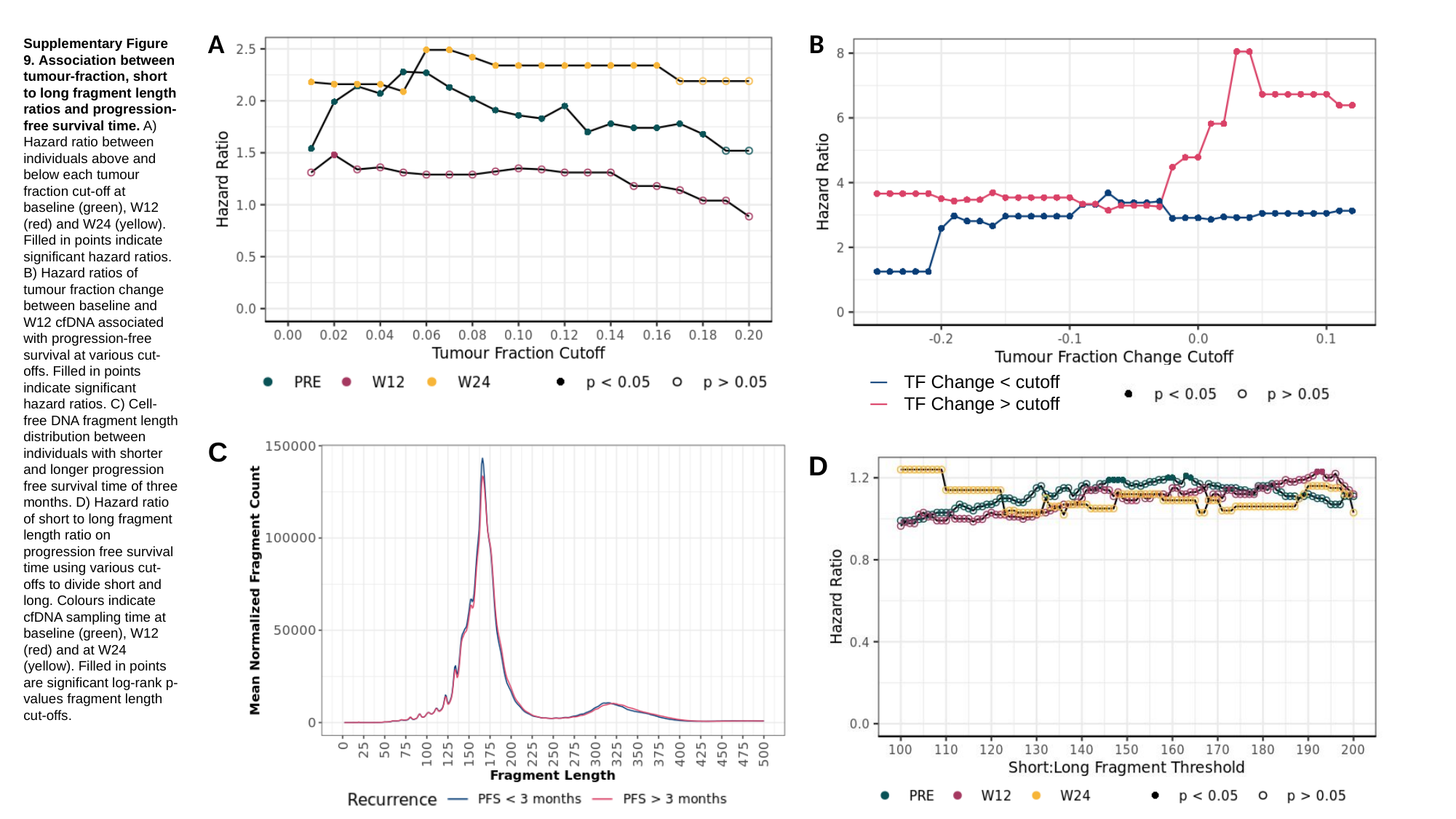

A
B
Supplementary Figure 9. Association between tumour-fraction, short to long fragment length ratios and progression-free survival time. A) Hazard ratio between individuals above and below each tumour fraction cut-off at baseline (green), W12 (red) and W24 (yellow). Filled in points indicate significant hazard ratios. B) Hazard ratios of tumour fraction change between baseline and W12 cfDNA associated with progression-free survival at various cut-offs. Filled in points indicate significant hazard ratios. C) Cell-free DNA fragment length distribution between individuals with shorter and longer progression free survival time of three months. D) Hazard ratio of short to long fragment length ratio on progression free survival time using various cut-offs to divide short and long. Colours indicate cfDNA sampling time at baseline (green), W12 (red) and at W24 (yellow). Filled in points are significant log-rank p-values fragment length cut-offs.
TF Change < cutoff
TF Change > cutoff
C
D
