# Supplementary material for: Continuous versus Standard Palbociclib Treatment and Molecular Profiling of Solid Tissues and Liquid Biopsies in the CCTG MA.38 Trial in Advanced Breast Cancer
Source: Cancer Res Commun. 2025 Nov 13;5(11):1998–2011. doi: 10.1158/2767-9764.CRC-25-0346 (PMC12613153; doi:10.1158/2767-9764.CRC-25-0346)
Supplement: Supplementary Table 1 — Breast Cancer 360 Gene Signatures and Description [file crc-25-0346_supplementary_table_1_suppst1.docx]

**Supplementary Table 1: Breast Cancer 360 Gene Signatures and Description**

| **Signature** | **Genes** | **Description** |
| --- | --- | --- |
| APM | TAP1, TAP2, TAPBP, HLA-A, HLA-B, HLA-C | Tumour Immunogenicity Antigen presenting (or processing) machinery. This signature measures the abundance of genes in the MHC Class I antigen presentation pathway and some key genes involved in processing the antigens prior to presentation. Typically, antigens from the cell cytoplasm are presented on Class I and recognized by the TCR on cytolytic CD8+ T cells. MHC Class I is expressed by all nucleated cells in the body, but downregulation of Class I MHC pathways is an evasion strategy that can be employed by tumour cells. An effective anti-tumour immune response depends on cytolytic T cells encountering neoantigens presented on the tumour cell surface. Strong anti-tumour immune responses are typically accompanied by high expression of antigen presentation genes. |
| Apoptosis | AXIN1, BAD, BAX, BBC3, BCL2L1, | Tumour Regulation This signature captures genes associated with apoptotic processes, specifically with genes involved in mitochondrial membrane integrity. It includes both pro- and anti-apoptotic genes. |
| AR | AR | Breast Cancer Receptors This gene is a type of nuclear receptor that is activated by binding any of the androgenic hormones. AR is widely expressed in breast cancer and has been shown to characterize a distinct molecular subset of triple negative breast cancer (TNBC) and suggested as a potential target candidate in this form of breast cancer. |
| B7-H3 | CD276 | Inhibitory Immune Mechanisms B7-H3 (CD276) gene expression. B7-H3 is a negative regulator of T cell activity that is expressed on both tumour and immune cells. |
| Basal | ACTR3B, ANLN, BAG1, BCL2, BIRC5, BLVRA, CCNB1, CCNE1, CDC20, CDC6, NUF2, CDH3, CENPF, CEP55, CXXC5, EGFR, ERBB2, ESR1, EXO1, FGFR4, FOXA1, FOXC1, GPR160, GRB7, KIF2C, NDC80, KRT14, KRT17, KRT5, MAPT, MDM2, MELK, MIA, MKI67, MLPH, MMP11, MYBL2, MYC, NAT1, ORC6, PGR, PHGDH, PTTG1, RRM2, SFRP1, SLC39A6, TMEM45B, TYMS, UBE2C, UBE2T | Basal-Like Breast Cancer Subtyping Basal-like tumours are typically characterized as having low expression of ER, PR, and HER2. Most clinically triple negative tumours are Basal-like subtype by molecular profiling. These tumours are poorly differentiated invasive high-grade ductal carcinomas that by have metastatic properties. |
| BC p53 | DDB2, CDKN1A, RNF103, APH1B, TCEAL1, BTG2, KIAA0040, MAP2K4, NPEPPS, GATA3, SLC39A6, CCND1, NEO1, TOP2A, CDC25C, NUDT1, FNBP1, CENPF, POLD1, NCAPH2, PTTG1, CDCA7L, GGH, CDK1, MKI67, KIFC1, MYBL2, KIF23, CEP55, UBE2C, CDC25B, TRIP13, MAD2L1, ATAD2, FOXM1, AURKA, CDKN3, CCNA2, TAP1, CKS1B, MCM3, TUBA4A, PREP, RFC4 | BC p53 Tumour Mutational Response This signature categorizes p53 status by mutant-like vs wild-type-like in breast cancer and the signature is significantly associated with overall survival in breast cancer, identifying a group with high unmet need. |
| BC Proliferation | MKI67, CEP55, KIF2C, MELK, CENPF, EXO1, ANLN, RRM2, UBE2C, CCNB1, CDC20 | BC Proliferation Tumour Regulation This signature outputs the PAM50 proliferation score by measuring key genes involved in breast tumour proliferation. In some cases, a highly proliferative breast tumour may correlate with an increase in disease progression or metastasis. |
| BRCAness | ADM, ANLN, ASPM, ATP10B, BBOX1, BLM, BRCA1, BTG2, SKA3, ISM1, C5orf38, CCNA2, CCNB1, CCNE1, CD68, CDC7, CDCA8, CDKN1C, CHRNA5, CRYAB, CYP4F3, DCN, DDX39A, DEPDC1, EXO1, FAM83D, FLRT3, FXYD3, GJB2, HIST1H1C, HLA-E, IL1R2, KCNB1, KIF14, KRT6B, LEMD1, MCM2, MCM3, MME, MT1G, NEIL3, NETO2, PCNA, PKMYT1, POLD1, POLQ, RFC4, RRM2, SPC25, ST6GALNAC2 | BRCAness Tumour Mutational Response This signature captures breast cancer biology that is informative as to defects in the DNA damage repair-genes BRCA1 and BRCA2. Similar to the Homologous Recombination Deficiency signature this captures breakdown in DNA damage repair, however, these are specific to BRCA-related mutations and more heavily weighted to BRCA1 mutants. |
| CD8 T-Cells | CD8A, CD8B | CD8 T-Cells Immune Cell Abundance This signature measures the abundance of CD8+ T cells in the tumour microenvironment. |
| CDK4 Expression | CDK4 | CDK4 Expression Breast Cancer Signaling Pathways Cyclin-dependent kinases 4 and 6 (CDK4/6) play a key role in the regulation of proliferation in normal breast tissue and breast tumours. CDK4/6 inhibitors have been indicated in hormone receptor (HR) positive metastatic breast cancer. Cyclin-dependent kinase 4 (CDK4) is an enzyme encoded by the CDK4 gene, mutations in this gene as well as in its related proteins have been shown to be associated with tumourigenesis. |
| CDK6 Expression | CDK6 | CDK6 Expression Breast Cancer Signaling Pathways Cyclin-dependent kinases 4 and 6 (CDK4/6) play a key role in the regulation of proliferation in normal breast tissue and breast tumours. CDK4/6 inhibitors have been indicated in hormone receptor (HR) positive metastatic breast cancer. CDK6, as well as CDK4, has been shown to phosphorylate and regulate the activity of the tumour suppressor protein Retinoblastoma and indicating a role in cancer development. |
| Cell Adhesion | CLDN1, CLDN3, CLDN4, CLDN7, OCLN, CDH1 | Cell Adhesion Tumour Regulation Epithelial cells use tight junction complexes to adhere to each other. Some breast cancers have greatly down-regulated expression of one or more of the genes coding for tight junction proteins. This phenomenon is common in claudin-low breast cancers, but it is not confined to that subtype. This signature scores samples for down-regulation in any of these tight junction genes. |
| Claudin-Low | CLDN3, CLDN4, CLDN7, SNAI1, SNAI2, TWIST1, ZEB2, CD44, ITGA6, ITGB1, MUC1, THY1, ADAM12, ADM, CDC20, DEPDC1, HES1, HLA-DPB1, MMP3, PIK3CD, TGFB1, ZFPM2 | Claudin-Low Breast Cancer Subtyping This molecular subtype is characterized by low levels of luminal differentiation markers, high enrichment for epithelial-to-mesenchymal transition markers, immune response and cancer stem cell-like genes. |
| Cytotoxic Cells | CTSW, GNLY, GZMA, GZMB, GZMH, KLRK1, PRF1, NKG7 | Cytotoxic Cells Immune Cell Abundance This signature measures the abundance of cytotoxic cells in the tumour microenvironment. Cytotoxic cells such as natural killer (NK) and CD8+ T cells use a number of molecules, including perforin, granzymes and killer cell lectin-like receptor (KLRG) family members to recognize, penetrate and kill infected cells. Cytotoxic activity is the mechanism by which the immune system most effectively kill tumour cells. |
| Cytotoxicity | GZMA, GZMB, GZMH, PRF1, GNLY | Cytotoxicity Anti-Tumour Immune Activity This signature measures the molecules used by natural killer (NK) and CD8+ T cells to mount a cytolytic attack on tumour cells. Cytotoxic cells such as NK and CD8+ T cells, use a number of molecules, including perforin, granzymes and granulysin to penetrate and kill infection cells and tumours. Cytotoxic activity is the mechanism by which the immune system most effectively kills tumour cells. |
| Differentiation | TSPAN1, AGR2, FSTL1, CLDN3, CLDN7, VCAN, CXADR, ELF3, FHL1, CBLC, ITGB6, MUC1, CEACAM6, DDR2, S100A14, HEG1, JAM2, SFRP1, BMP7, SLPI, SNAI2, TCF4, TMPRSS2, VIM, WNT5A, CAV1, GDF15, CDH1, | Differentiation Tumour Regulation This signature assigns a score of differentiation to the sample. Well-differentiated tumours that is phenotypically more similar to normal cells or tissue will grow and spread at a slow rated compared with poorly differentiated tumours, these present with abnormal cells that often grow rapidly. |
| Endothelial Cells | BCL6B, CDH5, CLEC14A, CXorf36, FAM124B, KDR, MMRN2, MYCT1, PALMD, ROBO4, TIE1 | Endothelial Cells Stromal Factors This signature measures genes associated with vascular tissue and angiogenesis. Angiogenesis is important for nutrient trafficking to the tumour and proper oxygenation for tumour growth. Tumour angiogenesis forms leaky inefficient vessels that can reduce efficiency of lymphocyte trafficking to tumours. |
| ER Signaling | ANXA9, BORCS7, ESR1, DNAJC12, TBC1D9, MAPT, NAT1, SCUBE2, SYTL4, TCEAL1, TFF1, ADCY9, ADD1, CDCA8, DDX39A, EIF3B, ELOVL2, HEMK1, IFT140, ITPR1, PFDN2, PGR, PTGER3, SERBP1, SHMT2, TLE3, WDR77 | ER Signaling Breast Cancer Signaling Pathways Estrogen-binding systems associate with various proteins that direct cell cycle signaling, proliferation and survival. This signature captures ER-mediated signaling pathways to elucidate how ER modulates activity of key transcription factors through stabilizing DNA-protein complexes and recruiting co-activators. This signature also captures the impact to other signaling pathways induced by the binding of estrogens in the nuclear causing conformational changes in the receptors. |
| ERBB2 | ERBB2 | ERBB2 Breast Cancer Receptors This gene encodes a member of the EGF receptor family of receptor tyrosine kinases. This protein has no ligand binding domain of its own and therefore cannot bind growth factors. However, it does bind tightly to other ligand-bound EGF receptor family members to form a heterodimer, stabilizing ligand binding and enhancing kinase-mediated activation of downstream signaling pathways. Amplification and overexpression are well established in breast cancer and the associated protein is a key pathological marker. |
| ESR1 | ESR1 | ESR1 Breast Cancer Receptors This gene encodes an estrogen receptor, a ligand-activated transcription factor composed of several domains important for hormone binding, DNA binding, and activation of transcription. The associated ER protein is a key pathological marker of breast cancer. |
| FOXA1 | FOXA1 | FOXA1 Tumour Regulation This transcription factor is involved in the regulation of gene expression in differentiated tissues. Sometimes associated with BRCA1 through cell cycle regulation. Also involved in ESR-1 mediated transcription and required for ESR1 binding to the NKX2-1 promoter in breast cancer. |
| Genomic Risk | ACTR3B, ANLN, BAG1, BCL2, BIRC5, BLVRA, CCNB1, CCNE1, CDC20, CDC6, NUF2, CDH3, CENPF, CEP55, CXXC5, EGFR, ERBB2, ESR1, EXO1, FGFR4, FOXA1, FOXC1, GPR160, GRB7, KIF2C, NDC80, KRT14, KRT17, KRT5, MAPT, MDM2, MELK, MIA, MKI67, MLPH, MMP11, MYBL2, MYC, NAT1, ORC6, PGR, PHGDH, PTTG1, RRM2, SFRP1, SLC39A6, TMEM45B, TYMS, UBE2C, UBE2T | Genomic Risk Breast Cancer Prognosis The Genomic Risk of Recurrence score (Genomic Risk) is calculated by comparing the expression profiles of 46 genes in the sample with the four PAM50 centroids, to calculate four different correlation values. These correlation values are then combined with the PAM50 proliferation score to estimate the genomic risk of distant recurrence. The results are reported on a scale of 0 to 100, with 0 being lowest risk and 100 being highest risk. This score is distinct from the Risk of Recurrence (ROR) score, as it does not include the tumour size included in the score calculation – it is solely based on the genomic data. |
| HER2E | ACTR3B, ANLN, BAG1, BCL2, BIRC5, BLVRA, CCNB1, CCNE1, CDC20, CDC6, NUF2, CDH3, CENPF, CEP55, CXXC5, EGFR, ERBB2, ESR1, EXO1, FGFR4, FOXA1, FOXC1, GPR160, GRB7, KIF2C, NDC80, KRT14, KRT17, KRT5, MAPT, MDM2, MELK, MIA, MKI67, MLPH, MMP11, MYBL2, MYC, NAT1, ORC6, PGR, PHGDH, PTTG1, RRM2, SFRP1, SLC39A6, TMEM45B, TYMS, UBE2C, UBE2T | HER2-E Breast Cancer Subtyping HER2-Enriched tumours are typically characterized as clinically HER2 positive breast cancer as defined by traditional IHC/FISH criteria. Some studies have indicated that the HER2-Enriched molecular subtype may be a better predictor of response to HER2-targeted therapies when compared with IHC and FISH. |
| HRD | ATP10B, BLM, BRCA1, BTG2, C5orf38, CCNE1, CDCA5, CDCA8, CHRNA5, CRYAB, FXYD3, HIST1H1C, HLA-E, KIF14, LEMD1, NETO2, RFC4, TRIP13 | HRD Tumour Mutational Response This signature is used to functionally assess Homologous Recombination Repair status, with potential to predict sensitivity to DNA-damage repair inhibitors such as PARP inhibitors. This captures cell cycle regulation, DNA damage, DNA replication, and DNA recombination and repair pathways. Additionally, this signature is also used to predict overall survival in breast cancer. |
| Hypoxia | ADM, BNIP3, PGK1, SLC2A1 | Hypoxia Inhibitory Metabolism This signature measures genes associated with reduced oxygenation in the tumour. Hypoxia can induce expression of many cancer promoting processes (e.g. invasion, motility, metabolic reprogramming) and can promote resistance to immune cell-mediated cytolysis and reduced cytolytic activity in natural killer (NK) and CD8+ T cells. |
| IDO1 | IDO1 | IDO1 Inhibitory Immune Mechanisms Indoleamine 2,3-dioxygenase 1 gene expression. IDO1 is expressed by tumour, immune, and stromal cells and is the rate-limiting enzyme of tryptophan catabolism. By catalyzing the degradation of tryptophan, which is necessary for cytolytic T cell proliferation and activity, IDO1 inhibits anti-tumour immune responses. |
| IFN Gamma | STAT1, CXCL9, CXCL10 | IFN Gamma Anti-Tumour Immune Activity This signature tracks the canonical response to type II interferon, including the most universal components of that response. IFNγ induces macrophage and natural killer (NK) cell activation, increases antigen presentation, and induces gene transcription patterns that can lead to immune cell recruitment to the tumour. IFNγ signaling expression is associated with response to anti-PD1/L1 therapy. |
| Inflammatory Chemokines | CCL2, CCL3L1, CCL4, CCL7, CCL8 | Inflammatory Chemokines Inhibitory Immune Signaling Inflammatory chemokines recruit both myeloid and lymphoid populations to the tumour microenvironment. |
| LumA | ACTR3B, ANLN, BAG1, BCL2, BIRC5, BLVRA, CCNB1, CCNE1, CDC20, CDC6, NUF2, CDH3, CENPF, CEP55, CXXC5, EGFR, ERBB2, ESR1, EXO1, FGFR4, FOXA1, FOXC1, GPR160, GRB7, KIF2C, NDC80, KRT14, KRT17, KRT5, MAPT, MDM2, MELK, MIA, MKI67, MLPH, MMP11, MYBL2, MYC, NAT1, ORC6, PGR, PHGDH, PTTG1, RRM2, SFRP1, SLC39A6, TMEM45B, TYMS, UBE2C, UBE2T | Breast Cancer Subtyping Luminal A tumours are typically characterized by high expression of estrogen receptor (ER), progesterone receptor (PR), and genes associated with ER activation1. These tumours are low-grade, tend to grow slowly, exhibit low expression of genes associated with cell cycle activation and have the best prognosis. |
| LumB | ACTR3B, ANLN, BAG1, BCL2, BIRC5, BLVRA, CCNB1, CCNE1, CDC20, CDC6, NUF2, CDH3, CENPF, CEP55, CXXC5, EGFR, ERBB2, ESR1, EXO1, FGFR4, FOXA1, FOXC1, GPR160, GRB7, KIF2C, NDC80, KRT14, KRT17, KRT5, MAPT, MDM2, MELK, MIA, MKI67, MLPH, MMP11, MYBL2, MYC, NAT1, ORC6, PGR, PHGDH, PTTG1, RRM2, SFRP1, SLC39A6, TMEM45B, TYMS, UBE2C, UBE2T | Breast Cancer Subtyping Luminal B tumours are typically characterized by high expression of estrogen receptor (ER), progesterone receptor (PR), and genes associated with ER activation1. These tumours tend to grow slightly faster than Luminal A tumours, exhibit high expression of genes associated with cell cycle activation and proliferation, and have a slightly worse prognosis than Luminal A tumours. |
| Macrophages | CD163, CD68, CD84 | Macrophages Immune Cell Abundance This signature measures the abundance of macrophages in the tumor microenvironment. Macrophages can either augment tumor immunity (e.g. by presenting antigen) or suppress tumor immunity (e.g. by releasing immunosuppressive cytokines). |
| Mammary Stemness | VIM, DDR2, SNAI2, VCAN, FSTL1, HEG1, FHL1, CAV1, JAM2, TCF4 | Mammary Stemness Tumor Regulation This signature measures a cluster of epithelial-to-mesenchymal transition (EMT) genes that are up-regulated in tumours with stem-cell-like expression profiles. Higher signature scores indicate more stem-like tumours. |
| Mast Cells | MS4A2, CPA3, HDC, TPSAB1 | Mast Cells Immune Cell Abundance This signature measures the abundance of mast cells in the tumour microenvironment. |
| MHC2 | HLA-DPA1, HLA-DPB1, HLA-DQB1, HLA-DRA, HLA-DRB1, HLA-DMA | MHC2 Anti-Tumour Immune Activity This signature measures the major human leukocyte antigens (HLA) involved in MHC Class II antigen presentation. Professional antigen presenting cells (dendritic cells, macrophages and B cells) use the class II MHC to present extracellular antigens to CD4+ T cells. Activation of CD4+ T cells induces expression of cytokines that can promote cytotoxic T cell activation and effective anti-tumour adaptive immune responses. Presence of MHC Class II molecules is associated with improved patient outcome. |
| PAM50 | ACTR3B, ANLN, BAG1, BCL2, BIRC5, BLVRA, CCNB1, CCNE1, CDC20, CDC6, NUF2, CDH3, CENPF, CEP55, CXXC5, EGFR, ERBB2, ESR1, EXO1, FGFR4, FOXA1, FOXC1, GPR160, GRB7, KIF2C, NDC80, KRT14, KRT17, KRT5, MAPT, MDM2, MELK, MIA, MKI67, MLPH, MMP11, MYBL2, MYC, NAT1, ORC6, PGR, PHGDH, PTTG1, RRM2, SFRP1, SLC39A6, TMEM45B, TYMS, UBE2C, UBE2T | PAM50 Breast Cancer Subtyping This 50-gene signature measures a gene expression profile that allows for the classification of breast cancer into four biologically distinct subtypes (Luminal A, Luminal B, HER2-Enriched, Basal-like). |
| PD-1 | PDCD1 | PD-1 Inhibitory Immune Signaling Program cell death receptor 1 gene expression. Program cell death receptor 1 (PD-1, PDCD1, CD279) is expressed predominantly on lymphocytes. It is upregulated upon activation and becomes a negative regulator of activation by preventing proliferation and cytokine secretion. PD-1 expression has been shown to be associated with tumour-specific T cells. |
| PD-L1 | CD274 | PD-L1 Inhibitory Immune Mechanisms Program cell death ligand 1 gene expression. Program cell death ligand 1 (PD-L1, CD274) is a ligand for PD-1 and negative regulator of T cell activity that is expressed on both tumour and immune cells. |
| PD-L2 | PDCD1LG2 | PD-L2 Inhibitory Immune Signaling Program cell death ligand 2 gene expression. Program cell death ligand 2 (PD-L2, PDCDLG2, CD273) is a ligand for PD-1 and negative regulator of T cell activity that is expressed on antigen-presenting cells. |
| PGR | PGR | PGR Breast Cancer Receptors This gene encodes a member of the steroid receptor superfamily. The encoded protein mediates the physiological effects of progesterone, which plays a central role in reproductive events and the associated protein is a key pathological marker of breast cancer. |
| PTEN | PTEN | PTEN Breast Cancer Signaling Pathways Phosphatase and tensin homolog gene expression. PTEN is a tumour suppressor gene that functions through the regulation of the Akt/PKB signaling pathway. Mutations or loss of PTEN expression are common across a range of cancer types, including breast cancer. |
| Rb1 | RB1 | Rb1 Tumour Regulation Retinoblastoma protein gene expression. RB is involved in cell cycle regulation and tumour progression. RB gene loss is occurs predominantly in triple negative breast cancer, as a result of homozygous deletion. |
| SOX2 | SOX2 | SOX2 Tumour Regulation SRY (sex determining region Y)-box 2 transcription factor gene expression. SOX2 regulates a number of critical processes in breast cancer including cell proliferation and metastasis. SOX2 expression has been shown to be associated with the prognosis of metastatic rtumours and disease recurrence. |
| Stroma | FAP, COL6A3, ADAM12, OLFML2B, PDGFRB, LRRC32 | Stroma Stromal Factors This signature measures stromal components in the tumour microenvironment. The tumour stroma is the collection of non-cancerous and nonimmune tissue components surrounding the tumour. Stroma can act as a physical barrier that excludes immune cells from the tumour, preventing effective anti-tumour immunity even when tumour-associated antigens have induced immune cell priming and activation. These cells can also secrete important signals to the tumour, affecting tumour biology and response to the immune system. |
| TGF-Beta | TGFB1 | TGF-Beta Inhibitory Immune Mechanisms Transforming Growth Factor Beta gene expression. TGFβ (TGFB1) is a pleotropic cytokine which inhibits anti-tumour immune activity and promotes tumour growth and survival. |
| TIGIT | TIGIT | TIGIT Inhibitory Immune Signaling T cell immunoreceptor and Ig and ITIMS gene expression. T cell immunoreceptor and Ig and ITIMS domains (TIGIT) is an immune checkpoint molecule that suppresses anti-tumour immune activity in CD8+ T cells and NK cells. |
| TIS | CCL5, CD27, CD274, CD276, CD8A, CMKLR1, CXCL9, CXCR6, HLA-DQA1, HLA-DRB1, HLA-E, IDO1, LAG3, NKG7, PDCD1LG2, PSMB10, STAT1, TIGIT | TIS Anti-Tumour Immune Activity Tumour Inflammation Signature. TIS measures the abundance of a peripherally suppressed adaptive immune response within the tumour.  This signature is trained to predict response to anti-PD1 therapy (pembrolizumab). It consists of genes related to Interferon gamma signaling (IFNγ), antigen presentation, natural killer (NK) and T cells and inhibitory pathways. It also consists of normalization genes that have been selected to give consistent expression levels across most tissue or tumour types. This signature is useful for predicting response to anti-PD1 therapy and determining hot and cold immune status across multiple cancer types. |
| Treg | FOXP3 | Treg Immune Cell Abundance Regulatory T cell abundance. Treg is measured by gene expression of Forkhead box P3 (FOXP3). FOXP3 is the canonical transcription factor that defines the regulatory T cell (Treg) population and is used to measure Treg abundance. Regulatory T cells suppress other T cell activities through a variety of mechanisms. |
