# Supplementary material for: Continuous versus Standard Palbociclib Treatment and Molecular Profiling of Solid Tissues and Liquid Biopsies in the CCTG MA.38 Trial in Advanced Breast Cancer
Source: Cancer Res Commun. 2025 Nov 13;5(11):1998–2011. doi: 10.1158/2767-9764.CRC-25-0346 (PMC12613153; doi:10.1158/2767-9764.CRC-25-0346)
Supplement: Supplementary Table 2 — Palbociclib Drug Exposure [file crc-25-0346_supplementary_table_2_suppst2.docx]

**Supplementary Table 2. Palbociclib Drug Exposure**

|  | **Palbociclib 100 mg (N=90)** | **Palbociclib 125 mg (N=89)** | **Total (N=179)** |
| --- | --- | --- | --- |
| **Median daily dose (mg)** | **87** | **125** | **100** |
| Cumulative dose (mg) |  |  |  |
| Mean | 23629 | 26430 | 25022 |
| Std | 17646 | 17582 | 17621 |
| Median | 19325 | 27500 | 22100 |
| Min | 500 | 2250 | 500 |
| Max | 66600 | 60250 | 66600 |
| **Dose intensity (mg/week)** |  |  |  |
| Mean | 555.7 | 580.8 | 568.2 |
| Std | 123.3 | 85.76 | 106.7 |
| Min | 269.2 | 320.5 | 269.2 |
| Max | 700 | 660.4 | 700 |
| **90% or more dose intensity: N(%)** | 37 (41.1) | 48 (53.9) | 85 (47.5) |
| **At least one dose modification**  **(delay, omission or reduction): N(%)** | 86 (95.6) | 80 (89.9) | 166 (92.7) |
| **Reason for dose delay or omission: N(%)** |  | | |
| Administrative | 15 (16.7) | 14 (15.7) | 29 (16.2) |
| Neutropenia | 63 (70.0) | 36 (40.4) | 99 (55.3) |
| Patient request | 18 (20.0) | 22 (24.7) | 40 (22.3) |
|  |  |  |  |
| Patient forgot | 32 (35.6) | 39 (43.8) | 71 (39.7) |
| Other Hematological Toxicity | 4 (4.4) | 2 (2.2) | 6 (3.4) |
| Other Biochemical Toxicity | 3 (3.3) | 9 (10.1) | 11 (6.1) |
| Fever/Infection | 11 (12.2) | 6 (6.7) | 17 (9.5) |
| Mucositis | 3 (3.3) | 4 (4.5) | 7 (3.9) |
| Fatigue | 2 (2.2) | 5 (5.6) | 7 (3.9) |
| Febrile Neutropenia | 3 (3.3) | 2 (2.2) | 5 (2.8) |
| Nausea/Vomiting | 3 (3.3) | 3 (3.4) | 6 (3.4) |
| Other Clinical Event | 44 (48.9) | 62 (69.7) | 106 (59.2) |
| **At least one dose reduction: N(%)** | 62 (68.9) | 25 (28.1) | 87 (48.6) |
| **Reason for dose reduction: N(%)** |  | | |
| Neutropenia/Granulocytopenia | 57 (63.3) | 13 (14.6) | 70 (39.1) |
| Other Clinical Event | 6 (6.7) | 13 (14.6) | 19 (10.6) |
| **Discontinuation: N(%)** | 67 (74.4) | 70 (78.7) | 137 (76.5) |
| **Reason for discontinuation: N(%)** |  | | |
| Death | 1 (1.1) | 1 (1.1) | 2 (1.1) |
| Progressive Disease (objective) | 56 (62.2) | 61 (68.5) | 117 (65.4) |
| Adverse events related to protocol therapy | 6 (6.7) | 4 (4.5) | 10 (5.6) |
| Patient Refusal (not related to adverse event) | 3 (3.3) | 1 (1.1) | 4 (2.2) |
| Symptomatic Progression | 1 (1.1) | 3 (3.4) | 4 (2.2) |
