# Supplementary material for: Continuous versus Standard Palbociclib Treatment and Molecular Profiling of Solid Tissues and Liquid Biopsies in the CCTG MA.38 Trial in Advanced Breast Cancer
Source: Cancer Res Commun. 2025 Nov 13;5(11):1998–2011. doi: 10.1158/2767-9764.CRC-25-0346 (PMC12613153; doi:10.1158/2767-9764.CRC-25-0346)
Supplement: Supplementary Table 3 — Incidence Adverse Events (greater than 20% of patients for non-hematological AEs and greater than 10% for haematological/ chemistry AEs) [file crc-25-0346_supplementary_table_3_suppst3.docx]

**Supplementary Table 3. Incidence Adverse Events (> 20% of patients for non-hematological AEs and >10% for haematological/ chemistry AEs)**

|  | **Palbociclib 100 mg (N=90)** | | | | | | | **Palbociclib 125 mg (N=89)** | | | | | | |  |
| --- | --- | --- | --- | --- | --- | --- | --- | --- | --- | --- | --- | --- | --- | --- | --- |
|  | **Grade*** | | | |  |  | %R** | **Grade*** | | | |  |  | **%R**** |  |
| **ADVERSE EVENT** | **1** | **2** | **3** | **4** | **TOTAL** | **(%)** | **R_PAL** | **1** | **2** | **3** | **4** | **TOTAL** | **(%)** | **R_PAL** | **P value***** |
| Neutrophils | 3 | 19 | 55 | 7 | 84 | 93 | 93 | 2 | 37 | 42 | 5 | 86 | 97 | 97 | 0.50 |
| Fatigue | 45 | 29 | 3 | 0 | 77 | 86 | 34 | 37 | 32 | 4 | 0 | 73 | 82 | 42 | 0.55 |
| Anemia | 52 | 20 | 3 | 0 | 75 | 83 | 83 | 46 | 14 | 5 | 0 | 65 | 73 | 73 | 0.11 |
| Platelets | 43 | 1 | 2 | 1 | 47 | 52 | 52 | 48 | 5 | 1 | 1 | 55 | 62 | 62 | 0.23 |
| Nausea | 35 | 12 | 0 | 0 | 47 | 52 | 24 | 37 | 10 | 1 | 0 | 48 | 54 | 31 | 0.88 |
| Diarrhea | 31 | 7 | 3 | 0 | 41 | 46 | 19 | 27 | 8 | 2 | 0 | 37 | 42 | 18 | 0.65 |
| Anorexia | 24 | 14 | 0 | 0 | 38 | 42 | 19 | 18 | 7 | 0 | 0 | 25 | 28 | 6 | 0.06 |
| Headache | 24 | 8 | 2 | 0 | 34 | 38 | 12 | 19 | 13 | 0 | 0 | 32 | 36 | 10 | 0.88 |
| Alopecia | 28 | 4 | 0 | 0 | 32 | 36 | 18 | 21 | 3 | 0 | 0 | 24 | 27 | 18 | 0.26 |
| Serum Creatinine | 21 | 6 | 0 | 0 | 27 | 30 | 30 | 17 | 2 | 1 | 0 | 20 | 22 | 22 | 0.31 |
| Vomiting | 20 | 6 | 0 | 0 | 26 | 29 | 10 | 17 | 4 | 2 | 0 | 23 | 26 | 9 | 0.74 |
| Mucositis oral | 16 | 5 | 2 | 0 | 23 | 26 | 21 | 16 | 7 | 1 | 0 | 24 | 27 | 19 | 0.87 |
| Dysgeusia | 17 | 1 | 0 | 0 | 18 | 20 | 10 | 10 | 3 | 0 | 0 | 13 | 15 | 8 | 0.43 |
| Upper respiratory infection | 0 | 18 | 0 | 0 | 18 | 20 | 8 | 0 | 23 | 0 | 0 | 23 | 26 | 11 | 0.38 |
| Rash maculo-papular | 17 | 1 | 0 | 0 | 18 | 20 | 7 | 14 | 5 | 1 | 0 | 20 | 22 | 12 | 0.72 |
| Epistaxis | 13 | 0 | 0 | 0 | 13 | 14 | 12 | 11 | 0 | 0 | 0 | 11 | 12 | 4 | 0.83 |
| Bilirubin | 4 | 5 | 1 | 1 | 11 | 12 | 12 | 5 | 0 | 1 | 0 | 6 | 7 | 7 | 0.31 |

* Adverse events graded according to CTCAE Version 4.0

**Considered by investigator to be 'possibly', 'probably' or 'definitely' related to protocol treatment

***Fisher exact test p-value for the total number of adverse events.
